# Regulatory Mechanism of the Atypical AP-1-Like Transcription Factor Yap1 in Cryptococcus neoformans

**DOI:** 10.1128/mSphere.00785-19

**Published:** 2019-11-20

**Authors:** Yee-Seul So, Shinae Maeng, Dong-Hoon Yang, Hyelim Kim, Kyung-Tae Lee, Seong-Ryong Yu, Jennifer L. Tenor, Vinay K. Giri, Dena L. Toffaletti, Samantha Arras, James A. Fraser, John R. Perfect, Yong-Sun Bahn

**Affiliations:** a Department of Biotechnology, Center for Fungal Pathogenesis, College of Life Science and Biotechnology, Yonsei University, Seoul, South Korea; b Department of Medicine, Division of Infectious Diseases, Duke University School of Medicine, Durham, North Carolina, USA; c Australian Infectious Diseases Research Centre, School of Chemistry and Molecular Biosciences, University of Queensland, Brisbane, Queensland, Australia; d Department of Molecular Genetics and Microbiology, Duke University School of Medicine, Durham, North Carolina, USA; Carnegie Mellon University

**Keywords:** AP-1-like transcription factor, *C. neoformans*, Mpk1, Yap1

## Abstract

AP-1-like transcription factors play evolutionarily conserved roles as redox sensors in eukaryotic oxidative stress responses. In this study, we aimed to elucidate the regulatory mechanism of an atypical yeast AP-1-like protein, Yap1, in the stress response and virulence of Cryptococcus neoformans. *YAP1* expression was induced and involved not only by oxidative stresses, such as H_2_O_2_ and diamide, but also by other environmental stresses, such as osmotic and membrane-destabilizing stresses. Yap1 was distributed throughout both the cytoplasm and the nucleus under basal conditions and more enriched within the nucleus in response to diamide but not to other stresses. Deletion of the C-terminal cysteine-rich domain (c-CRD), where the nuclear export signal resides, increased nuclear enrichment of Yap1 under basal conditions and altered resistance to oxidative stresses but did not affect the role of Yap1 in other stress responses and cellular functions. As a potential upstream regulator of Yap1, we discovered that Mpk1 is positively involved, but Hog1 is mostly dispensable. Pleiotropic roles for Yap1 in diverse biological processes were supported by transcriptome data showing that 162 genes are differentially regulated by Yap1, with further analysis revealing that Yap1 promotes cellular resistance to toxic cellular metabolites produced during glycolysis, such as methylglyoxal. Finally, we demonstrated that Yap1 plays a minor role in the survival of C. neoformans within hosts.

**IMPORTANCE** The human meningitis fungal pathogen, Cryptococcus neoformans, contains the atypical yeast AP-1-like protein Yap1. Yap1 lacks an N-terminal cysteine-rich domain (n-CRD), which is present in other fungal Yap1 orthologs, but has a C-terminal cysteine-rich domain (c-CRD). However, the role of c-CRD and its regulatory mechanism remain unknown. Here, we report that Yap1 is transcriptionally regulated in response to oxidative, osmotic, and membrane-destabilizing stresses partly in an Mpk1-dependent manner, supporting its role in stress resistance. The c-CRD domain contributed to the role of Yap1 only in resistance to certain oxidative stresses and azole drugs but not in other cellular functions. Yap1 has a minor role in the survival of C. neoformans in a murine model of systemic cryptococcosis.

## Abstract (precis)

The human meningitis fungal pathogen, Cryptococcus neoformans, contains the atypical yeast AP-1-like protein Yap1. Yap1 lacks an N-terminal cysteine-rich domain (n-CRD), which is present in other fungal Yap1 orthologs, but has a C-terminal cysteine-rich domain (c-CRD). However, the role of c-CRD and its regulatory mechanism remain unknown. Here, we report that Yap1 is transcriptionally regulated in response to oxidative, osmotic, and membrane-destabilizing stresses partly in an Mpk1-dependent manner, supporting its role in stress resistance. The c-CRD domain contributed to the role of Yap1 only in resistance to certain oxidative stresses and azole drugs but not in other cellular functions. Yap1 has a minor role in the survival of C. neoformans in a murine model of systemic cryptococcosis.

## INTRODUCTION

Sensing, responding, and adapting to a myriad of environmental stresses are key abilities for all living organisms to survive in their biological niches (1, 2). These capabilities are particularly important for infectious microbes that encounter dramatic changes in external conditions during colonization and proliferation within a host. For instance, Cryptococcus neoformans, a human meningoencephalitis fungal pathogen, can adapt to diverse environmental conditions, surviving and proliferating in both natural environments and other eukaryotic hosts (3, 4).

Among host-derived stresses, oxidative stresses generated by host phagocytic cells play pivotal roles in hampering the initial survival and proliferation of fungal pathogens (1, 5). For example, infectious propagules (spores or dried yeasts) of C. neoformans are inhaled through the upper respiratory tract to lung alveoli, which are under surveillance by resident alveolar macrophages. Cryptococcus neoformans produces two antiphagocytic factors, a polysaccharide capsule and a polyphenol melanin pigment, which prevent the pathogen from being readily phagocytosed (3, 4). Even after phagocytosis, C. neoformans is able to survive and proliferate within macrophages by inhibiting phagosome maturation and to escape through exocytosis without killing host cells (6, 7). Because phagolysosomes within macrophages attack such intracellular pathogens with toxic reactive oxygen species (ROS) (8) including superoxide anion (O_2_^•−^), hydrogen peroxide (H_2_O_2_), and hydroxyl radical (OH^−^), C. neoformans is assumed to have both conserved and unique oxidative stress defense systems to counteract such harsh oxidative stresses. In support of this, deletion of *SOD1*, which encodes a cytosolic Cu, Zn-superoxide dismutase that converts O_2_^•−^ to H_2_O_2_, attenuates the virulence of C. neoformans (9). Although catalases (Cat1 to -4) and glutathione peroxidases (Gpx1 and Gpx2), which detoxify H_2_O_2_ and organic peroxides, respectively, are dispensable for the virulence of C. neoformans (10, 11), the thioredoxin peroxidase (peroxiredoxin) system is essential (12). Deletion of genes involved in the peroxiredoxin system and its recycling, including *TSA1* (peroxiredoxins), *TRX1* (thioredoxin), *TRR1* (thioredoxin reductase), and *SRX1* (sulfiredoxin), severely affects the growth and virulence of C. neoformans (12–14).

In C. neoformans, several signaling pathways are involved in the oxidative stress defense (1). These include two mitogen-activated protein kinases (MAPKs), Hog1 and Mpk1. Hog1 is a central MAPK of the high-osmolarity glycerol response (HOG) pathway. The HOG pathway consists of the two-component-like phosphorelay system and the Ssk2-Pbs2-Hog1 MAPK module (1, 15). Mpk1 is a central MAPK of the cell wall integrity pathway in association with Mkk1/2 MAPK kinase (MAPKK) and Bck1 MAPKK kinase (MAPKKK) but controls some of the key oxidative stress defense genes (16–18). Therefore, deletion of either *HOG1* or *MPK1* reduces oxidative stress resistance of C. neoformans (18, 19). Nevertheless, their downstream transcription factor (TF) networks remain poorly understood. One Hog1-dependent TF candidate is a basic leucine zipper (bZIP) protein, Atf1. Deletion of *ATF1* greatly reduces resistance to diverse oxidative stresses (20) but does not significantly affect the virulence of C. neoformans (21), suggesting that C. neoformans may employ other TFs to control oxidative stress responses and adaptations. Surprisingly, our recent systematic functional profiling of 155 TFs in C. neoformans revealed that 95 of them are positively or negatively involved in oxidative stress responses (22), suggesting that the pathogen employs diverse classes of TFs to counteract such stresses.

Among the many candidate TFs involved in oxidative stress response, CNAG_00239 drew our attention for two reasons. First, CNAG_00239 was the only TF to promote resistance to all four oxidants H_2_O_2_, *tert*-butyl hydroperoxide (tBOOH), diamide, and menadione (MND) (22). Second, our previous transcriptome analysis demonstrated that CNAG_00239 expression is induced more than 2-fold in response to oxidative stress (23). CNAG_00239 encodes a protein containing a bZIP domain and with a protein size (701 amino acids) comparable to the yeast activator protein (Yap) family, in particular Yap1, but with very limited homology. Yap proteins function in a variety of cellular processes, including growth, differentiation, apoptosis, cell migration, and redox balance, in diverse eukaryotes ranging from fungi to mammals (24–26).

Among the Yap family of proteins, the yeast Yap1 and its orthologs are the best characterized in fungi, including Pap1 in Schizosaccharomyces pombe and Cap1 in Candida albicans (27, 28). Yap1 proteins generally contain three distinct domains: a bZIP domain in the N terminus important for DNA binding and two cysteine-rich domains at the N and C termini (named n-CRD and c-CRD, respectively) for redox regulation. A nuclear export sequence (NES) is also found inside the c-CRD, with the activity of Yap1 modulated by its subcellular localization rather than transcriptional control. Under normal conditions, Yap1 is distributed to both the cytoplasm and the nucleus. In response to oxidative stresses such as peroxides and oxidants, Yap1 undergoes conformational changes through intramolecular disulfide formation among cysteine residues in the c-CRD or n-CRD that prevent the NES from being recognized by the nuclear export protein Crm1, which results in its nuclear accumulation. If the c-CRD containing the NES is removed or Crm1 is deleted, Yap1 becomes constitutively localized and active within the nucleus. The oxidized form of Yap1 is reduced back through a thioredoxin system and can be exported out of the nucleus. In S. pombe, Pap1 plays similar roles as the yeast Yap1 (see reviews in references [Bibr B27][Bibr B28][Bibr B30]).

**FIG 1:**
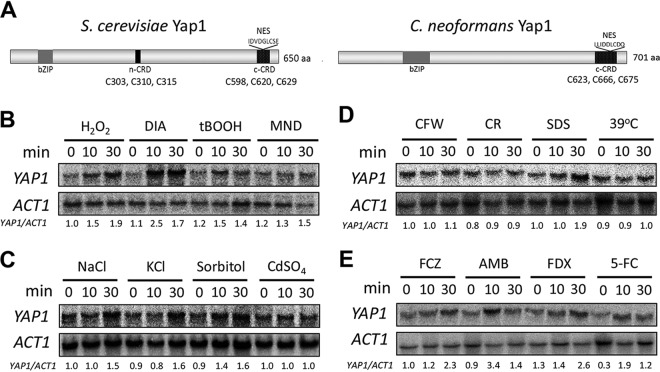
*YAP1* expression induced by various environmental stresses containing oxidative and cell membrane-destabilizing stresses. (A) Domain analysis of ScYap1 and CnYap1. (B to E) WT (H99) cells were grown to mid-logarithmic phase and exposed to various stress-inducing agents for the indicated time. Total RNA was isolated for Northern blot analysis. Expression levels of *YAP1* were detected with a radioactively labeled *YAP1*-specific probe. Relative expression levels of *YAP1* were quantified after normalization with *ACT1* expression levels. Northern blot analyses were repeated twice, with a representative result shown here. H_2_O_2_, 2.5 mM hydrogen peroxide; DIA, 2 mM diamide; tBOOH, 0.7 mM *tert*-butyl hydrogen peroxide; MND, 0.02 mM menadione; CFW, 3 mg/ml calcofluor white; CR, 0.8% Congo red; SDS, 0.03% sodium dodecyl sulfate; FCZ, 16 μg/ml fluconazole; AMB, 1 μg/ml amphotericin B; FDX, 3 μg/ml fludioxonil; 5-FC, 500 μg/ml flucytosine.

Conventional BLAST analysis of the yeast Yap1 sequence does not provide any interpretable hits in the *Cryptococcus* genome database, indicating that C. neoformans may employ an evolutionarily divergent type of Yap1-like TF. Supporting this, the CNAG_00239 protein appears to contain a Yap1 signature bZIP domain at the N terminus and a cysteine-rich domain at the C terminus (c-CRD) but lacks the n-CRD domain (Fig. 1A), suggesting that the n- and c-CRD-mediated conformational change may not occur. However, characteristic amino acid residues (Q239 and F242 in yeast Yap1) in bZIP domains distinguishing Yap proteins from the Gcn4-like AP-1 protein and NES within the c-CRD are highly conserved in the CNAG_00239 protein (Fig. 1A). Two other independent groups have recently reported on CNAG_00239 function, which has been named Yap1. Brown et al. reported that Yap1 promotes capsule production in C. neoformans (31). Paul et al. reported that Yap1 is required for oxidative stress responses to fluconazole treatment (32). These data for phenotypic *yap1*Δ mutant traits agree with our systematic *yap1*Δ mutant phenome data (22). Paul et al. reported that Yap1 is dispensable for the virulence of C. neoformans in a murine systemic cryptococcosis model (32). However, Jung et al. reported that Yap1 is required for virulence in an insect host model (22). Therefore, Yap1 may have a host-dependent role, albeit limited, in the virulence of C. neoformans. Regardless of these recent C. neoformans Yap1 reports, the role of c-CRD and its regulatory mechanism remains unknown. Here, we further characterize the role of c-CRD and its Yap1 regulatory mechanism, with a functional connection to the Hog1 and Mpk1 MAPK pathways in C. neoformans.

## RESULTS

### *YAP1* expression is induced by oxidative and membrane-destabilizing stresses.

To confirm *YAP1* expression patterns from our previous DNA microarray, we performed Northern blot analysis of *YAP1* under oxidative stress conditions. Accordingly, *YAP1* expression was induced in response to H_2_O_2_ within 10 min of exposure (Fig. 1B). In response to the thiol-specific oxidizing reagent diamide, *YAP1* expression was induced more within 10 min than H_2_O_2_ (Fig. 1B). In response to organic peroxide (*tert*-butyl hydroperoxide [tBOOH]) and superoxide generator (menadione [MND]), *YAP1* was also induced (Fig. 1B), albeit less than H_2_O_2_ and diamide. These data indicate that *YAP1* expression is induced by a broad range of oxidative stresses.

Next, we analyzed whether *YAP1* could be induced by other environmental stresses. In response to salt stresses (1 M NaCl or KCl), *YAP1* expression was induced within 30 min (Fig. 1C). *YAP1* expression was similarly induced by 1 M sorbitol (Fig. 1C), which may be correlated with membrane tension conferred by increased turgor pressure from osmotic stress. Supporting this, we also found that SDS, a membrane destabilizer, and fludioxonil, which stimulates overaccumulation of intracellular glycerols and cell swelling, induced *YAP1* expression within 30 min (Fig. 1D and E). *YAP1* expression was also induced by fluconazole and amphotericin B (AMB) (Fig. 1E), which affect membrane stability, and flucytosine (Fig. 1E), which inhibits DNA/RNA synthesis. In contrast, *YAP1* was not significantly induced by heavy metal (CdSO_4_), cell-wall-damaging agents (calcofluor white [CFW] and Congo red [CR]), or high temperature (Fig. 1C and D). In summary, *YAP1* expression is induced by both oxidative and membrane-destabilizing stresses.

### Yap1 promotes resistance to oxidative, osmotic, and membrane stresses and azole drugs.

**FIG 2:**
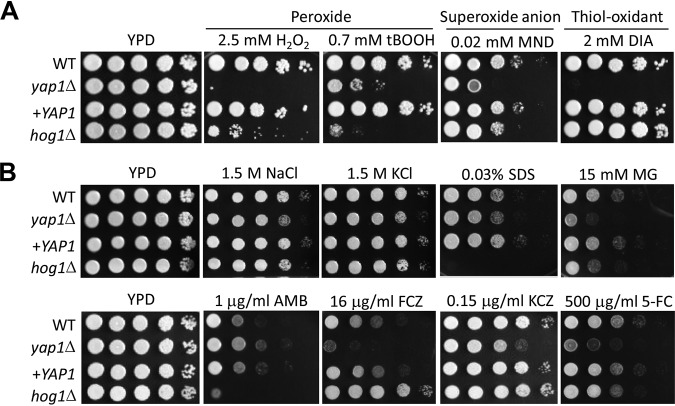
*YAP1* promotes cellular resistance to various environmental stresses. WT (H99) and *yap1*Δ (YSB815), *yap1*Δ::*YAP1* (YSB2122), and *hog1*Δ (YSB64) mutant strains were cultured in liquid YPD medium overnight at 30°C, 10-fold serially diluted, and spotted on YPD agar medium containing each stress inducer. Plates were further incubated at 30°C and photographed daily for 4 days. The spot assay was repeated more than three times with a representative image shown here. Stress inducers included peroxide (hydrogen peroxide [H_2_O_2_] and *tert*-butyl hydroperoxide [tBOOH]), superoxide anion (menadione [MND]), thiol oxidant (diamide [DIA]), methylglyoxal (MG), amphotericin B (AMB), fluconazole (FCZ), ketoconazole (KCZ), and flucytosine (5-FC).

Our previous functional profiling of C. neoformans TFs demonstrated that the *yap1*Δ mutant is susceptible to oxidants, osmotic stressors, and a membrane destabilizer and is highly susceptible to fluconazole and flucytosine (22, 32). To further confirm these data, we constructed the *yap1*Δ::*YAP1* complemented strain by reintroducing the wild-type (WT) copy of the *YAP1* gene. Verifying previous results, most Yap1-dependent phenotypes were completely recovered by the WT copy of *YAP1* (Fig. 2). The *yap1*Δ mutant was also highly sensitive to a toxic metabolite, methylglyoxal (Fig. 2B). Although phenotypic patterns of the *yap1*Δ mutant agreed with expression patterns of *YAP1* in general, there was one exception. *YAP1* expression was induced in response to AMB treatment (Fig. 1D), and *YAP1* deletion weakly increased resistance to AMB, which is in stark contrast to the finding that the *yap1*Δ mutant was more susceptible to azole drugs than the WT strain (Fig. 2B). This finding may imply that cells could sense the azole and polyene drug treatments as membrane stressors, subsequently inducing *YAP1*. In summary, Yap1 promoted resistance to oxidative stresses, osmotic and membrane stresses, toxic metabolites, and some antifungal drugs in C. neoformans.

### Cellular localization of Yap1 in C. neoformans.

Next, we addressed whether cellular localization of Yap1 is altered in response to environmental stresses in C. neoformans. Yap1 activity in budding yeast and Pap1 in fission yeast are mainly controlled by subcellular localization, rather than transcription, in response to oxidative stress or redox change (27, 28). Yap1/Pap1 subcellular localization is determined by the following factors: (i) conformational changes through disulfide bond formation between cysteine residues at the c-CRD and/or n-CRD, (ii) NES, and (iii) the NES-binding nuclear exportin Crm1. As previously described, C. neoformans Yap1 also contains the c-CRD and NES at its C terminus but lacks the n-CRD domain. To address whether C. neoformans Yap1 could be regulated by subcellular localization in addition to transcription control, we constructed a *yap1*Δ::*YAP1-GFP* strain by complementing the *yap1*Δ mutant with a GFP-tagged *YAP1* allele at the C terminus. Phenotypes of the *yap1*Δ::*YAP1-GFP* strain were equivalent to those of WT and *yap1*Δ::*YAP1* complemented strains (see Fig. S1 in the supplemental material), indicating that the Yap1-GFP protein is functional.

10.1128/mSphere.00785-19.1 FIG S1 Phenotypic analysis of *yap1*Δ::*YAP1* complemented and *yap1*Δ::*YAP1-GFP* strains. WT (H99), *hog1*Δ (YSB815), *yap1*Δ (YSB815), *yap1*Δ::*YAP1* (YSB2122), and *yap1*Δ::*YAP1-GFP* (YSB2723) strains were incubated overnight at 30°C, 10-fold serially diluted, and spotted on YPD agar medium containing each stress inducer. The plates were further incubated at 30°C and photographed daily for 4 days. This spot assay was repeated more than three times, with a representative image shown here. Download FIG S1, PDF file, 0.5 MB. Copyright © 2019 So et al. 2019 So et al. This content is distributed under the terms of the Creative Commons Attribution 4.0 International license.

**FIG 3:**
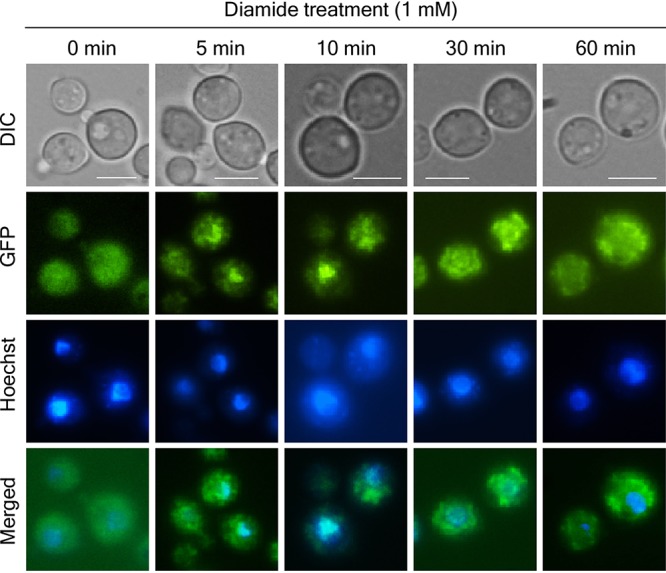
Cellular localization of *YAP1* in C. neoformans. The *yap1*Δ::*YAP1-GFP* (YSB2723) strain was grown to mid-log phase and treated with diamide. After diamide treatment, cells were further incubated at 30°C for the indicated times, fixed, and stained with Hoechst 33342 for nuclear visualization. Bar, 10 μm. DIC, differential interference contrast.

Because *YAP1* expression was strongly induced by diamide, we monitored cellular localization of Yap1-GFP upon diamide treatment (Fig. 3). Under untreated, basal conditions, Yap1-GFP localized to both the cytoplasm and the nucleus. Within 5 min of diamide treatment, Yap1-GFP was enriched in the nucleus and then redistributed evenly after 60 min (Fig. 3). However, such transient nuclear enrichment was not evident under other stress conditions (Fig. S2). Therefore, transient nuclear enrichment did not appear to be an essential role for Yap1 in other stress responses.

10.1128/mSphere.00785-19.2 FIG S2 Cellular localization of *yap1*Δ::*YAP1-GFP* and *yap1*Δ::*YAP1^c-CRDΔ^-GFP* strains under diverse environmental conditions. The *yap1*Δ::*YAP1-GFP* (YSB2723) and *yap1*Δ::*YAP1^c-CRD^*^Δ^*-GFP* (YSB5796) strains were exposed to H_2_O_2_, NaCl, SDS, fluconazole, or high temperature (39°C) for the indicated times. Cells were fixed and stained with Hoechst 33342 to visualize the nucleus. Bar, 10 μm. Download FIG S2, PDF file, 1.6 MB. Copyright © 2019 So et al. 2019 So et al. This content is distributed under the terms of the Creative Commons Attribution 4.0 International license.

### c-CRD and NES are critical for Yap1 function and localization in C. neoformans.

**FIG 4:**
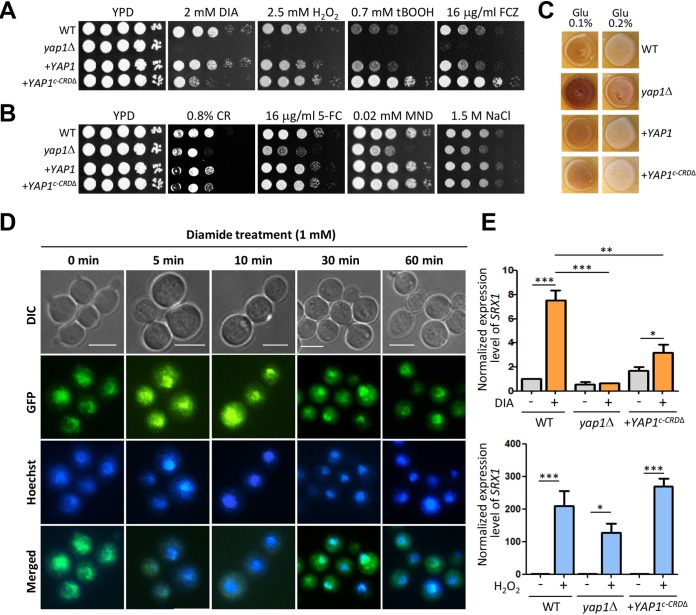
The cysteine-rich domain (c-CRD) only partially affects Yap1 function and cellular localization of CnYap1. (A and B) Strains (WT [H99], *yap1*Δ [YSB815], *yap1*Δ::*YAP1* [YSB2122], and *yap1*Δ::*YAP1^c-CRDΔ^-GFP* [YSB5796]) were spotted on YPD medium containing stress inducers. The plates were further incubated at 30°C and photographed daily for 4 days. This spot assay was repeated more than three times with a representative image shown here. DIA, diamide; H_2_O_2_, hydrogen peroxide; tBOOH, *tert*-butyl hydroperoxide; FCZ, fluconazole; CR, Congo red; 5-FC, flucytosine; MND, menadione. (C) For melanin production, WT (H99), *yap1*Δ (YSB815), *yap1*Δ::*YAP1* (YSB2122), and *yap1*Δ::*YAP1^c-CRDΔ^-GFP* (YSB5796) strains were incubated on Niger seed medium with either 0.1 or 0.2% glucose for 2 to 4 days at 37°C and photographed daily. These experiments were repeated twice with a representative result shown here. (D) The *yap1*Δ::*YAP1^c-CRDΔ^-GFP* (YSB5796) strain was treated with diamide (1 mM), and cellular localization was monitored. Hoechst stain was used to stain the nucleus. Bar, 10 μm. (E) Analysis of *SRX1* expression levels under 2 mM diamide or 2.5 mM H_2_O_2_ treatment conditions in WT (H99), *yap1*Δ (YSB815), and *yap1*Δ::*YAP1^c-CRDΔ^-GFP* (YSB5796) strains. cDNA was synthesized from total RNA extracted from the strains. Three independent biological experiments with three technical replicates were performed. Error bars indicate standard error of the mean. Statistical significance of the differences was determined by one-way analysis of variance with Bonferroni’s multiple-comparison test (*, *P* < 0.05; **, *P* < 0.001; ***, *P* < 0.0001).

Next, we addressed whether the c-CRD and NES are required for regulating Yap1 subcellular localization in C. neoformans. We constructed a *yap1*Δ::*YAP1^c-CRD^*^Δ^*-GFP* strain, where Yap1 lacking the c-CRD and NES regions (amino acids 623 to 676) was fused to GFP. Surprisingly, integration of the *YAP1^c-CRD^*^Δ^*-GFP* allele into the *yap1*Δ mutant generated complex phenotypes. First, the *yap1*Δ::*YAP1^c-CRD^*^Δ^*-GFP* strain was even more resistant to H_2_O_2_, tBOOH, and fluconazole than WT (Fig. 4A). However, integration of the *YAP1^c-CRD^*^Δ^*-GFP* allele only partially restored the diamide resistance in the *yap1*Δ mutant (Fig. 4A). In contrast, the *yap1*Δ::*YAP1^c-CRD^*^Δ^*-GFP* strain was as resistant to Congo red (CR), flucytosine, menadione, and salt stresses as WT (Fig. 4B), indicating that the c-CRD and NES domains are dispensable for responding and adapting to these stresses. Similarly, the increased melanin production of the *yap1*Δ mutant on Niger seed medium was restored to normal by the *YAP1^c-CRD^*^Δ^*-GFP* allele (Fig. 4C).

We then examined whether the c-CRD and NES domains are required for Yap1 transient nuclear enrichment. Even under basal, unstressed conditions, Yap1^c-CRDΔ^-GFP protein was more enriched in the nucleus (Fig. 4D). During diamide treatment, nuclear enrichment of Yap1^c-CRDΔ^-GFP protein was maintained (Fig. 4C). This altered Yap1^c-CRDΔ^-GFP localization under basal conditions may affect *yap1*Δ::*YAP1^c-CRD^*^Δ^*-GFP* resistance to H_2_O_2_, tBOOH, fluconazole, and diamide.

To explain why the *yap1*Δ::*YAP1^c-CRD^*^Δ^*-GFP* strain exhibited opposite resistance patterns to H_2_O_2_ and diamide, we examined induction of *SRX1*, which encodes a sulfiredoxin and promotes H_2_O_2_ and diamide resistance (14). As previously reported, H_2_O_2_ strongly induced *SRX1* expression (Fig. 4E). *SRX1* expression was also induced by diamide treatment, albeit to a lesser extent (Fig. 4D). *YAP1* deletion did not significantly affect H_2_O_2_-induced *SRX1* but abolished diamide induction (Fig. 4D). In the *yap1*Δ::*YAP1^c-CRD^*^Δ^*-GFP* strain, *SRX1* induction by diamide was only partly restored (Fig. 4E). This may explain why full diamide resistance was not restored in the *yap1*Δ::*YAP1^c-CRD^*^Δ^*-GFP* strain.

### *YAP1* expression is regulated in a Hog1-independent, partly Mpk1-dependent manner.

**FIG 5:**
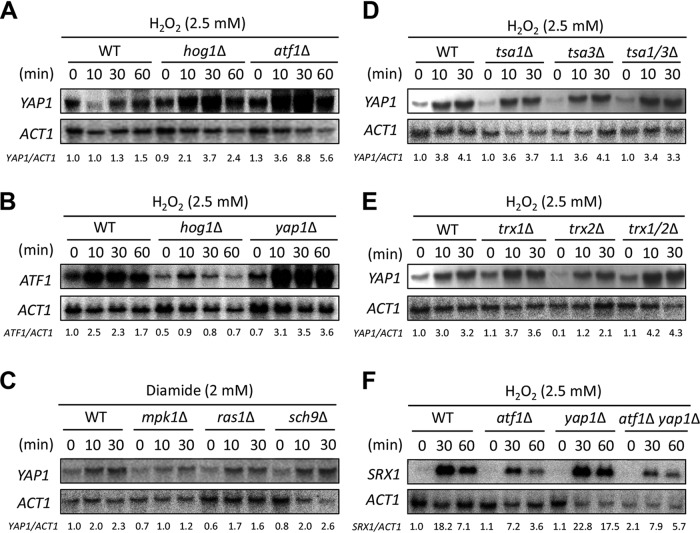
*YAP1* expression induced by various environmental stresses in a Hog1-independent but Mpk1-dependent manner. Strains (WT [H99], *hog1*Δ [YSB64], *atf1*Δ [YSB676], *yap1*Δ [YSB815], *mpk1*Δ [KK3], *ras1*Δ [YSB53], *sch9*Δ [YSB619], *tsa1*Δ [YSB1273], *tsa3*Δ [YSB1204], *tsa1*Δ *tsa3*Δ [YSB2735], *trx1*Δ [YSB1667], *trx2*Δ [YSB1791], *trx1*Δ *trx2*Δ [YSB1795], and *atf1*Δ *yap1*Δ [YSB4949]) were grown to mid-log phase and treated with 2.5 mM H_2_O_2_ or 2 mM diamide. Total RNA was isolated for Northern blot analysis. Each membrane was hybridized with gene-specific probes. The relative expression levels of *YAP1*, *ATF1*, and *SRX1* were quantitatively measured using a phosphorimager after normalization with *ACT1* expression levels (*YAP1*/*ACT1*, *ATF1*/*ACT1*, and *SRX1*/*ACT1*). These experiments were repeated twice with a representative image shown here.

We next sought the upstream signaling pathway responsible for *YAP1* regulation. First, we monitored Hog1 regulation of Yap1 because the HOG pathway is involved in the oxidative stress response. However, *HOG1* deletion induced *YAP1* expression more strongly in response to H_2_O_2_ (Fig. 5A), suggesting that Hog1 plays a distinct role from Yap1 in the oxidative stress response. Supporting this, the *hog1*Δ mutant was strongly diamide and azole drug resistant while the *yap1*Δ mutant displayed sensitivity (Fig. 2). Furthermore, in Atf1-deletion cells, which are defective downstream of Hog1, *YAP1* was also more strongly induced by H_2_O_2_ treatment (Fig. 5A). This suggested that inhibition of the HOG pathway may induce *YAP1* as a compensatory measure. If true, inhibition of *YAP1* would likely stimulate *ATF1* expression and *vice versa.* Indeed, *ATF1* was more strongly induced in the *yap1*Δ mutant than WT (Fig. 5B). Therefore, the Yap1-dependent signaling pathway may work independently from the HOG pathway to counteract oxidative stress responses and adaptions.

To identify a signaling component upstream of Yap1, we monitored *YAP1* expression in known stress-activated signaling pathways, including the Mpk1 MAPK, Ras-, and Sch9-dependent signaling pathways (1). Diamide-mediated *YAP1* induction was significantly reduced in the *mpk1*Δ mutant, although residual induction remained. In contrast, normal *YAP1* induction by diamide occurred in *ras1*Δ and *sch9*Δ mutants (Fig. 5C). Next, we addressed whether other oxidative stress defense systems, peroxiredoxins (Tsa1 and Tsa3) and thioredoxins (Trx1 and Trx2), are involved in *YAP1* induction. *YAP1* induction was normally induced even in the *tsa1*Δ *tsa3*Δ and *trx1*Δ *trx2*Δ double mutants (Fig. 5D and E). We also constructed an *atf1*Δ *yap1*Δ double mutant and monitored induction of the sulfiredoxin gene *SRX1*, a key oxidative stress response gene in C. neoformans (14) (Fig. 5F). *SRX1* induction was reduced in the *atf1*Δ mutant as expected from previous findings that the HOG pathway promotes *SRX1* induction (14) but was not reduced in the *yap1*Δ mutant (Fig. 5E). Deletion of *YAP1* did not further reduce *SRX1* induction in the *atf1*Δ mutant, suggesting that Yap1 is dispensable for *SRX1* induction in response to H_2_O_2_. Collectively, these data suggest that the Hog1-Atf1 and Mpk1-Yap1 pathways are two major signaling cascades to counteract oxidative stresses.

### Yap1 plays both Mpk1-dependent and -independent roles in C. neoformans.

**FIG 6:**
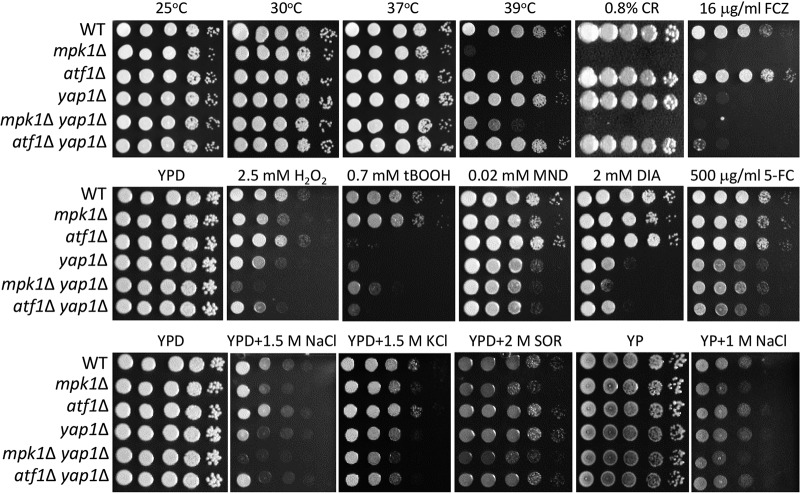
Yap1 and Atf1 play mostly independent roles in C. neoformans. Strains (WT [H99], *mpk1*Δ [KK3], *yap1*Δ [YSB815], *mpk1*Δ *yap1*Δ [YSB3092], and *atf1*Δ *yap1*Δ [YSB4949]) were cultured in liquid YPD medium overnight at 30°C, 10-fold serially diluted, and spotted on YPD or yeast extract-peptone (YP) medium containing stress-inducing agents. To test thermotolerance, spotted cells were incubated at 25°C, 30°C, 37°C, and 39°C and photographed daily for 3 days. CR, Congo red; FCZ, fluconazole; H_2_O_2_, hydrogen peroxide; tBOOH, *tert*-butyl hydroperoxide; MND, menadione; DIA, diamide; 5-FC, flucytosine; SOR, sorbitol.

The finding that Mpk1 regulates *YAP1* induction during oxidative stress prompted us to address their functional relationship. We constructed the *mpk1*Δ *yap1*Δ double mutant and compared its phenotypes with those of each single mutant (Fig. 6). Interestingly, the *mpk1*Δ *yap1*Δ mutant also exhibited thermosensitivity, albeit to a lesser extent than the *mpk1*Δ mutant (Fig. 6). The *mpk1*Δ *yap1*Δ mutant exhibited *mpk1*Δ mutant levels of sensitivity to fluconazole and osmotic stresses, whereas the *yap1*Δ mutant showed intermediate phenotypes (Fig. 6), suggesting that Yap1 may be one of several Mpk1 downstream TFs. In contrast, in response to diamide and oxidative stresses, the *yap1*Δ mutant showed higher sensitivity than the *mpk1*Δ mutant, with the *mpk1*Δ *yap1*Δ mutant exhibiting higher sensitivity than each single mutant (Fig. 6) and suggesting that Yap1 may be activated (or transcriptionally induced) by Mpk1 and another upstream regulator(s). The *mpk1*Δ, *yap1*Δ, and *mpk1*Δ *yap1*Δ mutants all exhibited similar levels of enhanced urease production (Fig. S3), implying that Mpk1 and Yap1 could be in a linear relationship under urease conditions. In summary, our data highlighted the complex relationship between Mpk1 and Yap1 under different growth conditions in C. neoformans.

10.1128/mSphere.00785-19.3 FIG S3 Effect of *YAP1* and *MPK1* genes on urease production. WT (H99), *mpk1*Δ (KK3), *yap1*Δ (YSB815), *yap1*Δ::*YAP1* (YSB2122), and *mpk1*Δ *yap1*Δ (YSB3092) strains were incubated overnight at 30°C and spotted on Christensen’s (urea-containing) agar medium. Plates were incubated at 30°C for 2 days and photographed daily. Experiments were performed twice, with a representative image shown here. Download FIG S3, PDF file, 0.5 MB. Copyright © 2019 So et al. 2019 So et al. This content is distributed under the terms of the Creative Commons Attribution 4.0 International license.

In addition, we compared the phenotypes of *yap1*Δ, *atf1*Δ, and *yap1*Δ *atf1*Δ mutants. Overall, the *yap1*Δ *atf1*Δ mutant was phenotypically indistinguishable from the *yap1* mutant (Fig. 6). Although we showed that *YAP1* and *ATF1* expression was more highly induced by H_2_O_2_ in *atf1*Δ and *yap1*Δ mutants, respectively (Fig. 5), the *yap1*Δ *atf1*Δ mutant was not more susceptible to oxidative stresses than each single mutant (Fig. 6). These data suggested that Yap1 and Atf1 play mostly independent roles in C. neoformans.

### Yap1-dependent transcriptome profiles.

**FIG 7:**
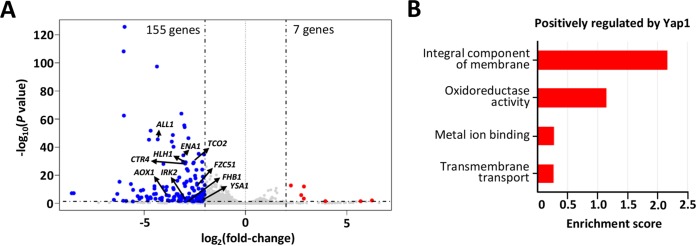
Yap1-regulated genes under basal conditions. RNA sequencing-based transcriptome analysis of WT and *yap1*Δ mutant strains was performed under basal conditions. (A) Volcano plot for gene expression changes. Dashed lines are cutoff values (|log_2_FC| > 2, *P < *0.05). (B) GO term analysis for the genes which were positively regulated by Yap1.

To elucidate the downstream networks governed by Yap1, we performed RNA sequencing (RNA-seq)-based transcriptome analysis of WT and *yap1*Δ mutant strains. Under basal conditions (yeast extract-peptone-dextrose [YPD] medium, 30°C), the expression of 155 genes was significantly reduced in the *yap1*Δ mutant, including *ALL1*, *AOX1*, *FHB1*, *CTR4*, *ENA1*, *YFH701*, *IRK2*, *TCO2*, *HLH1*, and *FZC51*, whereas the expression of only 7 genes was increased (|log_2_FC| > 2, *P < *0.05) (Fig. 7A and Data Set S1). The biological functions of some of these genes partly explain Yap1-dependent phenotypes. Based on the C. neoformans kinase and transcription factor phenome database that we previously reported (22, 33), Tco2, Hlh1, and Fzc51 are involved in H_2_O_2_, diamide, and *tert*-butyl hydroperoxide resistance, respectively. Ena1, a cation transporter, plays critical roles in cation homeostasis, pH regulation, membrane stability, and C. neoformans virulence (34, 35). When the Yap1-dependent genes were analyzed by Gene Ontology (GO) terms, genes positively regulated by Yap1 were mainly categorized into transmembrane helix, oxidoreductase activity, transmembrane transport, and metal ion binding (Fig. 7B and Data Set S1). In contrast, genes negatively regulated by Yap1 were not categorized by GO term analysis (Fig. 7B and Data Set S1). In conclusion, Yap1 likely serves as a transcriptional activator for a large number of genes regulating the oxidative stress response and membrane stability of C. neoformans.

10.1128/mSphere.00785-19.6 DATA SET S1 Yap1-dependent transcriptome profiles. Download Data Set S1, XLSX file, 0.7 MB. Copyright © 2019 So et al. 2019 So et al. This content is distributed under the terms of the Creative Commons Attribution 4.0 International license.

### Yap1 plays a minor role in the virulence of C. neoformans.

**FIG 8:**
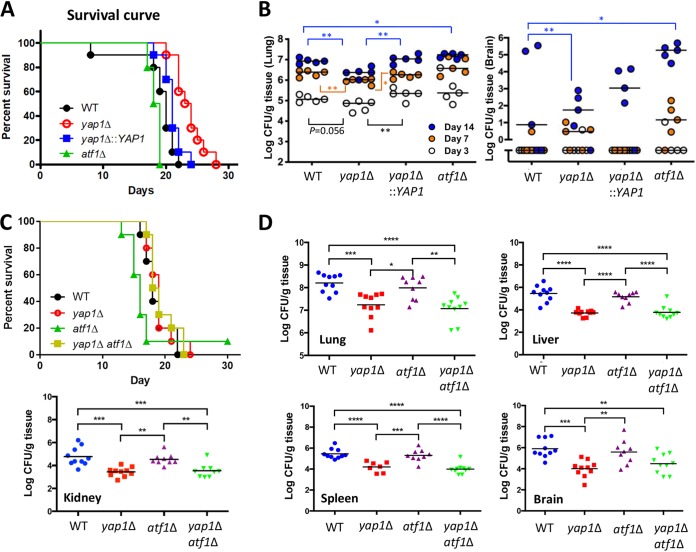
Yap1 plays a minor role in C. neoformans virulence. (A) Ten mice (A/J mice) were evaluated for the effect of two transcription factors on virulence. Statistics were calculated by log rank (Mantel-Cox) test, with *P* values as follows: WT versus *yap1*Δ mutant, 0.0002; WT versus *yap1*Δ::*YAP1* mutant, 0.0670; WT versus *atf1*Δ mutant, 0.0117; *yap1*Δ versus *yap1*Δ::*YAP1* mutant, 0.0066. (B) Fungal burden of lungs and brains from A/J mice infected with C. neoformans strains was monitored at days 3, 7, and 14 postinoculation. Five mice were evaluated per C. neoformans strain. The Mann-Whitney test was used to compare the fungal burdens of the different strains (*, *P* < 0.05; **, *P* < 0.01). (C) To further compare WT (H99), *yap1*Δ (YSB815), *atf1*Δ (YSB676), and *yap1*Δ *atf1*Δ (YSB2432) strain virulence, 10 BALB/c mice were infected by nasal inhalation. Kaplan-Meier survival curves were plotted using GraphPad Prism 7.0. Significance was analyzed using the log rank test, and *P* values of <0.05 were considered significant. (D) Fungal burdens of the lungs, liver, kidney, spleen, and brain were collected from BALB/c mice once body weight had decreased to 80% of preinfection weight. *P* values of <0.05 were considered significant (*, *P* < 0.05; **, *P* < 0.01; ***, *P* < 0.001; ****, *P* < 0.0001).

Previously, Paul et al. demonstrated that the *yap1*Δ mutant constructed in the KN99α strain background is as virulent as WT (32). In contrast, the *yap1*Δ mutant constructed in the H99 strain background is attenuated in virulence in an insect host model (22). Here, we further examined the role of Yap1 in C. neoformans virulence using a systemic cryptococcosis murine model. Similar to the results of Paul et al., the *yap1*Δ mutant was almost as virulent as WT and *yap1*Δ::*YAP1* complemented strains (Fig. 8A). However, the *yap1*Δ mutant also showed significantly reduced fungal burden in both lungs and brain compared to WT and *yap1*Δ::*YAP1* complemented strains (Fig. 8B). In contrast, the *atf1*Δ mutant was almost as virulent as WT (Fig. 8A).

We next addressed whether Yap1 and Atf1 play synergistic roles in C. neoformans virulence. The *yap1*Δ *atf1*Δ mutant was as virulent as WT and each single mutant strain. In agreement with *in vitro* phenotypes, however, the *yap1*Δ *atf1*Δ mutant showed reduced fungal burden like the *yap1*Δ mutant in all infected tissues (lungs, brains, kidneys, spleens, and liver) in the systemic cryptococcosis murine model. In conclusion, Yap1 plays a minor role in C. neoformans virulence, whereas Atf1 is mostly dispensable.

## DISCUSSION

**FIG 9:**
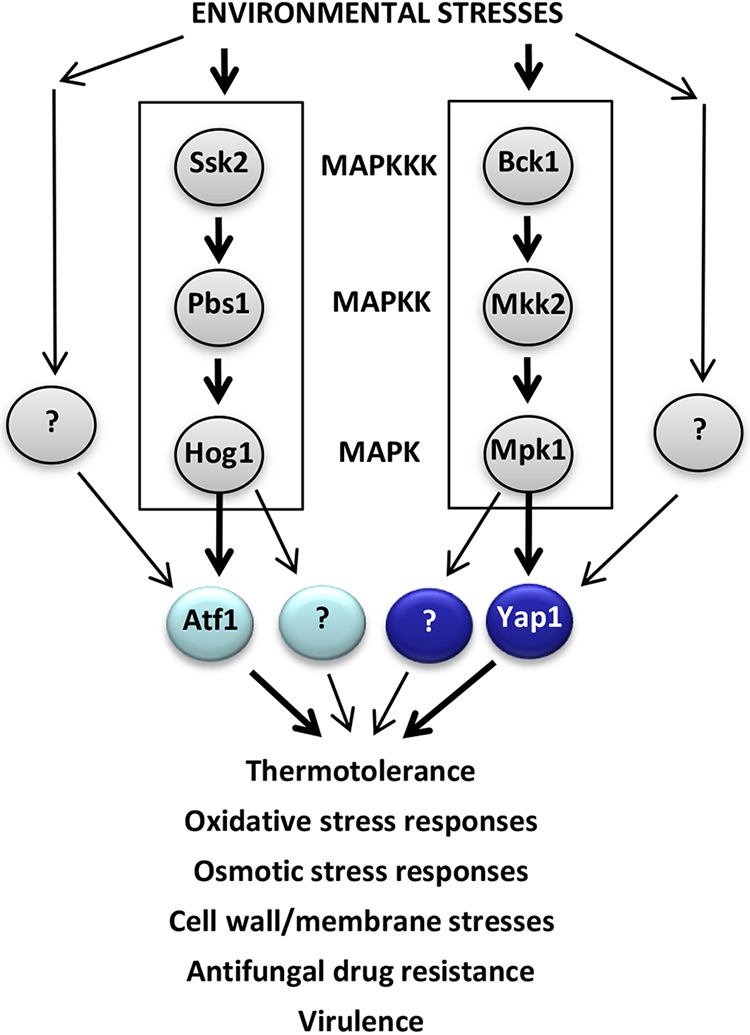
Schematic summary of Yap1-related signaling cascades under environmental stresses. Yap1 is the transcription factor working downstream of the Mpk1 MAPK pathway required for maintaining cell wall integrity and responding to oxidative stress responses, whereas Atf1 is the transcription factor working downstream of the Hog1 MAPK pathway required for responding to oxidative and general stresses. However, Yap1 and Atf1 are responsible for only a subset of Mpk1- and Hog1-dependent functions, respectively, and are also regulated by other signaling pathways. Therefore, Yap1 and Atf1 play distinct and shared roles in regulating a variety of stresses in C. neoformans.

In this study, we elucidated the regulatory mechanism of Yap1 in association with Hog1 and Mpk1 MAPK pathways for C. neoformans stress responses and pathogenicity. The proposed Atf1 and Yap1 regulatory mechanism in C. neoformans is summarized in Fig. 9. *ATF1* is induced by oxidative stresses mainly in a Hog1 MAPK-dependent manner, whereas *YAP1* is mainly regulated by the Mpk1 MAPK. Nevertheless, other upstream regulators may be implicated because *ATF1* and *YAP1* could be weakly induced in the absence of Hog1 or Yap1, respectively. Hog1 and Mpk1 likely have multiple other downstream TFs. The Hog1-Atf1 and Mpk1-Yap1 pathways are compensatory for each other because the absence of one MAPK pathway may trigger the activation of the other MAPK pathway in response to certain environmental stresses.

It has long been thought that C. neoformans lacks a Yap1-like TF. In BLAST searches for C. neoformans Yap orthologs using protein sequences of Saccharomyces cerevisiae Yap1 to Yap8 (ScYap1 to ScYap8), only two *Cryptococcus* proteins (CNAG_01242.2 and CNAG_00055.2) show very limited homology to ScYap4 with very low scoring (42.743, E value of 9.06535E^−5^), although both contain bZIP-like domains. Based on the reverse BLAST search with *Cryptococcus* proteins in the *Saccharomyces* genome database (SGD), CNAG_01242.2 appears more homologous to ScYap5 than ScYap4, whereas CNAG_00055.2 does not produce any hit. For other yeast Yap proteins, no meaningful hits were obtained at a cutoff E value of 0.001. However, increasing the cutoff E value to 0.1 yields several hits. Among these, two genes (CNAG_04630 and CNAG_00239) have a bZIP domain. Interestingly, the reverse BLAST SGD analysis showed that CNAG_00239.2 is significantly homologous to ScYap1 (score, 108; E value, 4.3E^−07^), whereas CNAG_04630.2 is homologous to ScYap5. Furthermore, BLAST searching with Pap1 in fission yeast (SpPap1) gives CNAG_00239 as a first hit. Therefore, overall homology of CNAG_00239 to ScYap1/SpPap1 is low, yet it contains a conserved Yap-signature bZIP domain at the N terminus and a cysteine-rich domain at the C terminus (c-CRD) with NES.

There are several reasons why we consider C. neoformans Yap1 (CnYap1) as an “atypical” AP-1-like bZIP TF. First, CnYap1 does not have any N-terminal cysteine residues required for H_2_O_2_-mediated intramolecular disulfide formation with the c-CRD that causes ScYap1 or SpPap1 to be accumulated in the nucleus through NES hiding in the c-CRD (see reviews in references 27 and 28). In both budding and fission yeasts, ScYap1/SpPap1 proteins serve as redox sensors, particularly for peroxide stress, and such conformational change is essential for ScYap1/Pap1 activity. Supporting this, H_2_O_2_ treatment of C. neoformans did not significantly enrich CnYap1 nuclear localization. During manuscript preparation, Paul et al. independently reported this gene (CNAG_00239) and named it *YAP1* (32). They demonstrated that heterologous expression of C. neoformans *YAP1* partly complements the phenotypes of the S. cerevisiae *yap1*Δ mutant (diamide and CdSO_4_ sensitivity but not H_2_O_2_ sensitivity), suggesting that CnYap1 retains part of ScYap1 function apparently stemming from the lack of n-CRD in CnYap1. However, regardless of the missing n-CRD, Paul et al. showed that CnYap1-GFP could be enriched in the nucleus when it is expressed in S. cerevisiae, proposing that the three cysteine residues in the c-CRD could be sufficient for CnYap1 nuclear enrichment in budding yeast. In S. cerevisiae, two cysteine residues within the C-terminal region (Cys598 and Cys620) are preferentially formed upon exposure to H_2_O_2_ (36). Our study demonstrates that c-CRD deletion increased Yap1 nuclear enrichment even under basal, unstressed conditions and rendered cells more resistant to H_2_O_2_ and tBOOH. However, c-CRD is dispensable for Yap1-mediated resistance to cell wall, flucytosine, menadione, and osmotic stresses. Therefore, it seems that c-CRD only partially affects CnYap1 function and cellular localization. Transient nuclear enrichment of Yap1 with diamide treatment could be controlled by other factors rather than the oxidation status of c-CRD cysteine residues. Another possibility is that Yap1 phosphorylation by Mpk1 and others controls its cellular localization and activity. In fact, in S. cerevisiae, Yap1 undergoes phosphorylation upon H_2_O_2_ exposure, which correlates with its nuclear enrichment, although the role of such phosphorylation is not clear (37). Whether CnYap1 undergoes oxidative stress-dependent phosphorylation through Mpk1 is under investigation.

The second “atypical” feature of CnYap1 is its upstream regulator. Control of CnYap1 expression by Mpk1 in C. neoformans is very unexpected, as a regulatory connection between Yap1/Pap1 and cell wall integrity MAPKs has not been observed or reported in budding and fission yeast models. ScYap1 and SpPap1 activity is known to be controlled by thioredoxin peroxidase (Tsa1 in S. cerevisiae) or glutaredoxin peroxidase (Gpx3 in S. pombe) (27, 28). Our study demonstrates that neither peroxiredoxins (Tsa1 and Tsa3) nor thioredoxins (Trx1 and Trx2) affect *YAP1* induction during oxidative stress. The distinct features of CnYap1 are also revealed in some phenotypic traits of its deletion mutant (e.g., fluconazole sensitivity), which are not conserved in other Yap1 mutants such as S. cerevisiae, S. pombe, C. albicans, Candida glabrata, and Aspergillus fumigatus (32).

Here, we demonstrate that Yap1 plays a minor role in the virulence of C. neoformans, and the additional deletion of *ATF1* does not further reduce virulence. Previously, Paul et al. reported that Yap1 is dispensable for C. neoformans KN99α strain virulence (32). Discrepancies between these studies may reside in a minor C. neoformans strain background difference (H99S here versus KN99α). In our two independent mouse studies (Fig. 8A and C), the *yap1*Δ mutant showed weakly reduced virulence in an A/J mouse model but normal virulence in a BALB/c mouse model, indicating that the role of Yap1 in C. neoformans virulence may depend on host systems. Nevertheless, *YAP1* deletion reduced fungal burden in both mouse models. A minor role of Yap1 itself in virulence, regardless of its pleiotropic roles, could be explained in that some virulence factors, such as melanin and ureases, are enhanced in the *yap1*Δ mutant. Therefore, Yap1 likely plays both positive and negative roles in promoting C. neoformans pathogenicity.

## MATERIALS AND METHODS

### Ethics statement.

The BALB/c mouse study was carried out in strict accordance with recommendations in the Australian Code of Practice for the Care and Use of Animals for Scientific Purposes by the National Health and Medical Research Council. The protocol was approved by the Molecular Biosciences Animal Ethics Committee (AEC) of The University of Queensland (AEC approval no. SCMB/010/17). Infection was performed under methoxyflurane anesthesia, and all efforts were made to minimize suffering through adherence to the Guidelines to Promote the Wellbeing of Animals Used for Scientific Purposes as put forward by the National Health and Medical Research Council (Australia). For the A/J mouse study, protocol A178-14-07 was reviewed and approved by the Duke University Institutional Animal Care and Use Committee (IACUC). All studies were performed in compliance with Duke University institutional guidelines for animal experimentation.

### Strains and media.

Table S1 in the supplemental material lists the strains used in this study. C. neoformans strains were cultured and maintained in yeast extract-peptone-dextrose (YPD) medium. Melanin production was assessed on Niger seed medium (70 g Niger seed, 20 g Bacto agar per liter) containing different glucose concentrations. Cells were incubated at 37°C and photographed daily for 1 to 3 days by microscope (SMZ-168; Motic) at ×10 magnification. For the urease assay, equal cell numbers (5 × 10^4^ cells) were spotted onto Christensen’s agar medium. Plates were incubated for 2 to 3 days at 30°C and photographed.

10.1128/mSphere.00785-19.4 TABLE S1 *Cryptococcus* strains used in this study. Download Table S1, DOCX file, 0.04 MB. Copyright © 2019 So et al. 2019 So et al. This content is distributed under the terms of the Creative Commons Attribution 4.0 International license.

### Construction of C. neoformans mutants.

The *YAP1* gene has been deleted in C. neoformans serotype A strain H99 (*MAT*α) (22). The disruption cassettes were generated by first-round PCR and second-round overlap PCR followed by biolistic transformation, as previously described (38). All PCR amplifications were performed using *Ex Taq* polymerase (TaKaRa). Transformants were selected on YPD containing nourseothricin, G-418, or hygromycin B. The *yap1*Δ mutants in different strain backgrounds were confirmed by diagnostic PCR and Southern blot analysis (22). The same strategy described above was used to delete *ATF1*, *HOG1*, and *MPK1* genes using primer sets described in Table S2.

10.1128/mSphere.00785-19.5 TABLE S2 Primers used in this study. Download Table S2, DOCX file, 0.02 MB. Copyright © 2019 So et al. 2019 So et al. This content is distributed under the terms of the Creative Commons Attribution 4.0 International license.

### Construction of *yap1*Δ::*YAP1-GFP* complemented and *yap1*Δ::*YAP1^c-CRD^*^Δ^*-GFP* strains.

To verify *yap1*Δ mutant phenotypes and resolve Yap1 localization, the *yap1*Δ::*YAP1-GFP* complemented strain was constructed. The full-length *YAP1* gene was amplified using PCR with primers listed in Table S2 and cloned into pTOP vector (Enzynomics) and sequenced. The *YAP1* gene was subcloned into pNEO-GFPht. Then, pNEO-YAP1-GFPht was transformed into the *yap1*Δ mutant strain (YSB815). All strains were confirmed with diagnostic PCR and spot assays. To generate *yap1*Δ::*YAP1^c-CRD^*^Δ^*-GFP*, 5′ and 3′ flanking regions of c-CRD of *YAP1* were amplified by PCR with primers listed in Table S2. The amplified 5′ and 3′ flanking regions were cloned into pTOP-V2 vector (Enzynomics) and sequenced. The flanking regions were subcloned into pNEO-GFPht to generate pNEO_YAP1^c-CRDΔ^-GFP. pNEO-YAP1^c-CRDΔ^-GFPht was transformed into the *yap1*Δ mutant strain (YSB815). All the strains were confirmed with diagnostic PCR.

### Total RNA isolation and Northern blot analysis.

Each strain was grown in 50 ml YPD medium at 30°C overnight. Strains were incubated in YPD liquid medium overnight at 30°C. The overnight culture was inoculated into 150 ml fresh YPD liquid medium and then incubated at 30°C until 600-nm optical density (OD_600_) reached approximately 0.6. The 50-ml culture was treated with 2.5 mM H_2_O_2_, 2 mM diamide, 0.08 mM *tert*-butyl hydroperoxide, 0.02 mM menadione, 5 mg/ml calcofluor white, 0.8% Congo red, 0.03% SDS, 1 M NaCl, 1 M KCl, 2 M sorbitol, 25 μM CdSO_4_, 16 μg/ml fluconazole, 1 μg/ml amphotericin B, 3 μg/ml fludioxonil, or 500 μg/ml flucytosine and then further incubated at 30°C for the times indicated in Fig. 1 and 5. For Northern blot analysis under temperature upshift conditions, cells were further incubated at 39°C. Cells were frozen in liquid nitrogen for 30 min and lyophilized. Total RNA was isolated by Easy-BLUE (iNtRON) (23). Expression levels of each gene were detected by Northern blotting analysis with gene-specific probes amplified by PCR using primers listed in Table S2.

### Stress sensitivity test.

Each strain was incubated in 2 ml liquid YPD medium overnight at 30°C, serially diluted (1 to 10^4^ dilutions) in distilled water (dH_2_O), and spotted onto solid medium containing each stress-inducing chemical indicated in Fig. 2, 4, and 6 and Fig. S2. Each plate was incubated and photographed daily for 2 to 4 days.

### Yap1 localization assay.

To monitor Yap1 subcellular localization, *yap1*Δ::*YAP1-GFP* and *yap1*Δ::*YAP1^c-CRDΔ^-GFP* strains were incubated overnight at 30°C in YPD medium. Cells were inoculated in fresh YPD medium and further incubated until OD_600_ reached approximately 0.6. Cells were treated with 1 mM diamide, 2.5 mM H_2_O_2_, 14 μg/ml fluconazole, or 0.03% SDS or exposed to 39°C and further incubated for 0 to 60 min. Treated cells were fixed with paraformaldehyde (4%) containing sucrose (3.4%) and incubated for 15 min at room temperature. Cells were washed with KPO_4_-sorbitol solution (1.2 M sorbitol and 0.1 M KPO_4_) and resuspended in small volumes of KPO_4_-sorbitol solution. For nuclear staining, Hoechst 33342 (Thermo Fisher) was added to fixed cells and incubated in the dark for 1 h. Cells were observed with an Olympus BX51 microscope.

### Transcriptome analysis by RNA sequencing.

WT (H99) and *yap1*Δ mutant (YSB815) strains were incubated in liquid YPD medium overnight at 30°C. Cells were subcultured in fresh YPD medium and further incubated at 30°C until OD_600_ reached 0.6. WT and *yap1*Δ mutant cells were harvested and lyophilized. Total RNA was isolated by Easy-BLUE (iNtRON) and purified with an RNeasy Mini kit (Qiagen). cDNA libraries were constructed with a TruSeq RNA library kit v2 (Illumina) using 1 μg total RNA and sequenced using the Illumina platform. Sequenced reads were trimmed to remove the adaptor sequence and masked for low-complexity or low-quality sequence using Cutadapt v2.4 with Python 3.5.2 with adaptor sequences i7 (AGATCGGAAGAGCACACGTCTGAACTCCAGTCAC) and i5 (AGATCGGAAGAGCGTCGTGTAGGGAAAGAGTGT). Reads were aligned to the genome sequence of C. neoformans var. *grubii* H99 strain retrieved from the Broad Institute (https://www.broadinstitute.org/fungal-genome-initiative/cryptococcus-neoformans-serotype-genome-project) using Hisat2 v2.1.0 with the Hisat and Bowtie 2 algorithm (39) and processed as previously reported (40). Briefly, Hisat2 was performed with “-p 30” and “–dta -1” options and other parameters set as default. Aligned reads were converted and sorted using SAMtools v0.1.19 with “-Sb -@ 8” for converting and “-@ 20 –m 2000000000” option for sorting and other parameters set as default. Transcript assembly and abundance estimation were performed by Stringtie v1.3.6 using “-p 12” option and also “-B” option to run the Ballgown. Assembled transcripts were merged to a single GTF file, and relative transcript abundances were displayed via FPKM (fragments per kilobase of exon per million fragments mapped). Read count matrix was generated by python script ‘prepDE.py’ and analyzed by DESeq2. Differential expression analysis was performed using DESeq2 v1.24.0 with default sets with the Ballgown. The volcano plot was illustrated using R v3.5.3 with the cutoff (|log_2_FC| > 2 with *P < *0.05). To analyze gene functional categories, we used DAVID Bioinformatics Resources 6.8 (NIAID/NIH).

### Mouse inhalation model.

To compare WT (H99), *yap1*Δ (YSB815), *yap1*Δ::*YAP1* (YSB2129), and *atf1*Δ (YSB676) strain virulence, 5 × 10^4^ cells were introduced via the nares into anesthetized (treated with 3% isoflurane via an induction box) 7- to 8-week-old female A/J mice (The Jackson Laboratory). Five mice were used to assess fungal load at days 3, 7, and 14 postinoculation, and 10 mice were used to assess survival. C. neoformans strains were grown in 5 ml YPD at 30°C overnight in 50-ml conical tubes at 220 rpm, washed twice with 10 ml PBS, and resuspended in 1 ml PBS. Cell counts were performed using the Cellometer Auto T4 cell counter (Nexcelom), and final concentrations were adjusted to 2 × 10^6^ CFU/ml such that a 25-μl dose introduced 5 × 10^4^ cells into each mouse. Inoculum concentrations were confirmed by plating dilutions on YPD plates. Animals were monitored daily. Mice were euthanized by carbon dioxide according to guidelines set by the Duke University Institutional Animal Care and Use Committee (IACUC). Fungal burden on lungs and brain for five mice was assessed at time of death. Organs were removed aseptically, weighed, and homogenized in 1 ml PBS prior to inoculation on YPD plates containing 100 μg/ml chloramphenicol. Plates were incubated at 30°C for 3 days, and CFU were enumerated. GraphPad software version 7 was used to generate the graphs and for the statistical analysis. Virulence assay protocols (protocol A178-14-07) were approved by the Duke University IACUC.

To further compare WT (H99), *yap1*Δ (YSB815), *atf1*Δ (YSB676), and *yap1*Δ *atf1*Δ (YSB2432) strain virulence, 6-week-old female BALB/c mice (Animal Resources Centre, Australia) were infected by nasal inhalation. For each strain, 10 mice were inoculated with 50 μl containing 5 × 10^5^ C. neoformans cells. No more than 5 mice were housed per individually ventilated cage (IVC) (Tecniplast, USA) with Bed-o’Cobs 1/8-in. bedding (The Andersons, USA), Crink-l’Nest nesting material (The Andersons, USA), and cardboard as environmental enrichment. Mice were provided Rat and Mouse Cubes (Specialty Feeds, Australia) and water *ad libitum*. Each mouse was examined and weighed twice daily for the experimental duration, with affected mice euthanized via CO_2_ inhalation once body weight had decreased to 80% of preinfection weight or they exhibited symptoms consistent with infection. Kaplan-Meier survival curves were plotted using GraphPad Prism 7.0. Significance was analyzed using the log rank test and *P* values of <0.05 were considered significant. Death was confirmed by pedal reflex prior to dissection. The brain, lungs, liver, spleen, and kidneys were collected, homogenized, and plated to determine CFU per gram organ weight. Kaplan-Meier survival curves were plotted using GraphPad Prism 7.0 (GraphPad Software, USA). Significance was analyzed using the log rank test, while organ burden significance was determined using a one-way ANOVA with Tukey’s multiple-comparison test. *P* values of <0.05 were considered significant.

### Data availability.

RNA-seq data are deposited in the Gene Expression Omnibus (GEO) database (accession number GSE136832). We will provide any materials used in this study upon request.

## References

[1] Bahn YS, Jung KW 2013 Stress signaling pathways for the pathogenicity of *Cryptococcus*. Eukaryot Cell 12:1564–1577. doi:10.1128/EC.00218-13. 24078305 PMC3889573

[2] Bahn YS, Xue C, Idnurm A, Rutherford JC, Heitman J, Cardenas ME 2007 Sensing the environment: lessons from fungi. Nat Rev Microbiol 5:57–69. doi:10.1038/nrmicro1578. 17170747

[3] Idnurm A, Bahn YS, Nielsen K, Lin X, Fraser JA, Heitman J 2005 Deciphering the model pathogenic fungus *Cryptococcus neoformans*. Nat Rev Microbiol 3:753–764. doi:10.1038/nrmicro1245. 16132036

[4] Kwon-Chung KJ, Fraser JA, Doering TL, Wang Z, Janbon G, Idnurm A, Bahn YS 2014 *Cryptococcus neoformans* and *Cryptococcus gattii*, the etiologic agents of cryptococcosis. Cold Spring Harb Perspect Med 4:a019760. doi:10.1101/cshperspect.a019760. 24985132 PMC4066639

[5] Brown DM, Hutchison L, Donaldson K, MacKenzie SJ, Dick CA, Stone V 2007 The effect of oxidative stress on macrophages and lung epithelial cells: the role of phosphodiesterases 1 and 4. Toxicol Lett 168:1–6. doi:10.1016/j.toxlet.2006.10.016. 17129690

[6] Johnston SA, May RC 2013 *Cryptococcus interactions* with macrophages: evasion and manipulation of the phagosome by a fungal pathogen. Cell Microbiol 15:403–411. doi:10.1111/cmi.12067. 23127124

[7] Garcia-Rodas R, Zaragoza O 2012 Catch me if you can: phagocytosis and killing avoidance by *Cryptococcus neoformans*. FEMS Immunol Med Microbiol 64:147–161. doi:10.1111/j.1574-695X.2011.00871.x. 22029633

[8] Nicola AM, Casadevall A, Goldman DL 2008 Fungal killing by mammalian phagocytic cells. Curr Opin Microbiol 11:313–317. doi:10.1016/j.mib.2008.05.011. 18573683 PMC2563425

[9] Cox GM, Harrison TS, McDade HC, Taborda CP, Heinrich G, Casadevall A, Perfect JR 2003 Superoxide dismutase influences the virulence of *Cryptococcus neoformans* by affecting growth within macrophages. Infect Immun 71:173–180. doi:10.1128/iai.71.1.173-180.2003. 12496163 PMC143417

[10] Giles SS, Stajich JE, Nichols C, Gerrald QD, Alspaugh JA, Dietrich F, Perfect JR 2006 The *Cryptococcus neoformans* catalase gene family and its role in antioxidant defense. Eukaryot Cell 5:1447–1459. doi:10.1128/EC.00098-06. 16963629 PMC1563583

[11] Missall TA, Cherry-Harris JF, Lodge JK 2005 Two glutathione peroxidases in the fungal pathogen *Cryptococcus neoformans* are expressed in the presence of specific substrates. Microbiology 151:2573–2581. doi:10.1099/mic.0.28132-0. 16079336

[12] Missall TA, Pusateri ME, Lodge JK 2004 Thiol peroxidase is critical for virulence and resistance to nitric oxide and peroxide in the fungal pathogen, *Cryptococcus neoformans*. Mol Microbiol 51:1447–1458. doi:10.1111/j.1365-2958.2004.03921.x. 14982637

[13] Missall TA, Lodge JK 2005 Thioredoxin reductase is essential for viability in the fungal pathogen *Cryptococcus neoformans*. Eukaryot Cell 4:487–489. doi:10.1128/EC.4.2.487-489.2005. 15701811 PMC549343

[14] Upadhya R, Kim H, Jung KW, Park G, Lam W, Lodge JK, Bahn YS 2013 Sulphiredoxin plays peroxiredoxin-dependent and -independent roles via the HOG signalling pathway in *Cryptococcus neoformans* and contributes to fungal virulence. Mol Microbiol 90:630–648. doi:10.1111/mmi.12388. 23998805 PMC3943550

[15] Bahn YS 2008 Master and commander in fungal pathogens: the two-component system and the HOG signaling pathway. Eukaryot Cell 7:2017–2036. doi:10.1128/EC.00323-08. 18952900 PMC2593196

[16] Gerik KJ, Bhimireddy SR, Ryerse JS, Specht CA, Lodge JK 2008 *PKC1* is essential for protection against both oxidative and nitrosative stresses, cell integrity, and normal manifestation of virulence factors in the pathogenic fungus *Cryptococcus neoformans*. Eukaryot Cell 7:1685–1698. doi:10.1128/EC.00146-08. 18689526 PMC2568057

[17] Kraus PR, Fox DS, Cox GM, Heitman J 2003 The *Cryptococcus neoformans* MAP kinase Mpk1 regulates cell integrity in response to antifungal drugs and loss of calcineurin function. Mol Microbiol 48:1377–1387. doi:10.1046/j.1365-2958.2003.03508.x. 12787363 PMC1635492

[18] Bahn YS, Geunes-Boyer S, Heitman J 2007 Ssk2 mitogen-activated protein kinase kinase kinase governs divergent patterns of the stress-activated Hog1 signaling pathway in *Cryptococcus neoformans*. Eukaryot Cell 6:2278–2289. doi:10.1128/EC.00349-07. 17951522 PMC2168243

[19] Bahn YS, Kojima K, Cox GM, Heitman J 2005 Specialization of the HOG pathway and its impact on differentiation and virulence of *Cryptococcus neoformans*. Mol Biol Cell 16:2285–2300. doi:10.1091/mbc.e04-11-0987. 15728721 PMC1087235

[20] Kim MS, Ko YJ, Maeng S, Floyd A, Heitman J, Bahn YS 2010 Comparative transcriptome analysis of the CO_2_ sensing pathway via differential expression of carbonic anhydrase in *Cryptococcus neoformans*. Genetics 185:1207–1219. doi:10.1534/genetics.110.118315. 20516494 PMC2927750

[21] Missall TA, Lodge JK 2005 Function of the thioredoxin proteins in *Cryptococcus neoformans* during stress or virulence and regulation by putative transcriptional modulators. Mol Microbiol 57:847–858. doi:10.1111/j.1365-2958.2005.04735.x. 16045626

[22] Jung KW, Yang DH, Maeng S, Lee KT, So YS, Hong J, Choi J, Byun HJ, Kim H, Bang S, Song MH, Lee JW, Kim MS, Kim SY, Ji JH, Park G, Kwon H, Cha S, Meyers GL, Wang LL, Jang J, Janbon G, Adedoyin G, Kim T, Averette AK, Heitman J, Cheong E, Lee YH, Lee YW, Bahn YS 2015 Systematic functional profiling of transcription factor networks in *Cryptococcus neoformans*. Nat Commun 6:6757. doi:10.1038/ncomms7757. 25849373 PMC4391232

[23] Ko YJ, Yu YM, Kim GB, Lee GW, Maeng PJ, Kim S, Floyd A, Heitman J, Bahn YS 2009 Remodeling of global transcription patterns of *Cryptococcus neoformans* genes mediated by the stress-activated HOG signaling pathways. Eukaryot Cell 8:1197–1217. doi:10.1128/EC.00120-09. 19542307 PMC2725552

[24] Bertini E, Oka T, Sudol M, Strano S, Blandino G 2009 YAP: at the crossroad between transformation and tumor suppression. Cell Cycle 8:49–57. doi:10.4161/cc.8.1.7259. 19106601

[25] Piccolo S, Dupont S, Cordenonsi M 2014 The biology of YAP/TAZ: hippo signaling and beyond. Physiol Rev 94:1287–1312. doi:10.1152/physrev.00005.2014. 25287865

[26] Toone WM, Morgan BA, Jones N 2001 Redox control of AP-1-like factors in yeast and beyond. Oncogene 20:2336–2346. doi:10.1038/sj.onc.1204384. 11402331

[B27] Boronat S, Domenech A, Paulo E, Calvo IA, Garcia-Santamarina S, Garcia P, Encinar Del Dedo J, Barcons A, Serrano E, Carmona M, Hidalgo E 2014 Thiol-based H_2_O_2_ signalling in microbial systems. Redox Biol 2:395–399. doi:10.1016/j.redox.2014.01.015. 24563858 PMC3926117

[B28] Herrero E, Ros J, Belli G, Cabiscol E 2008 Redox control and oxidative stress in yeast cells. Biochim Biophys Acta 1780:1217–1235. doi:10.1016/j.bbagen.2007.12.004. 18178164

[29] Antelmann H, Helmann JD 2011 Thiol-based redox switches and gene regulation. Antioxid Redox Signal 14:1049–1063. doi:10.1089/ars.2010.3400. 20626317 PMC3113447

[B30] Rodrigues-Pousada C, Menezes RA, Pimentel C 2010 The Yap family and its role in stress response. Yeast 27:245–258. doi:10.1002/yea.1752. 20148391

[31] Brown JCS, Nelson J, VanderSluis B, Deshpande R, Butts A, Kagan S, Polacheck I, Krysan DJ, Myers CL, Madhani HD 2014 Unraveling the biology of a fungal meningitis pathogen using chemical genetics. Cell 159:1168–1187. doi:10.1016/j.cell.2014.10.044. 25416953 PMC4243055

[32] Paul S, Doering TL, Moye-Rowley WS 2015 *Cryptococcus neoformans* Yap1 is required for normal fluconazole and oxidative stress resistance. Fungal Genet Biol 74:1–9. doi:10.1016/j.fgb.2014.10.015. 25445311 PMC4293237

[33] Lee KT, So YS, Yang DH, Jung KW, Choi J, Lee DG, Kwon H, Jang J, Wang LL, Cha S, Meyers GL, Jeong E, Jin JH, Lee Y, Hong J, Bang S, Ji JH, Park G, Byun HJ, Park SW, Park YM, Adedoyin G, Kim T, Averette AF, Choi JS, Heitman J, Cheong E, Lee YH, Bahn YS 2016 Systematic functional analysis of kinases in the fungal pathogen *Cryptococcus neoformans*. Nat Commun 7:12766. doi:10.1038/ncomms12766. 27677328 PMC5052723

[34] Idnurm A, Walton FJ, Floyd A, Reedy JL, Heitman J 2009 Identification of *ENA1* as a virulence gene of the human pathogenic fungus *Cryptococcus neoformans* through signature-tagged insertional mutagenesis. Eukaryot Cell 8:315–326. doi:10.1128/EC.00375-08. 19151325 PMC2653249

[35] Jung KW, Strain AK, Nielsen K, Jung KH, Bahn YS 2012 Two cation transporters Ena1 and Nha1 cooperatively modulate ion homeostasis, antifungal drug resistance, and virulence of *Cryptococcus neoformans* via the HOG pathway. Fungal Genet Biol 49:332–345. doi:10.1016/j.fgb.2012.02.001. 22343280 PMC3319253

[36] Kuge S, Arita M, Murayama A, Maeta K, Izawa S, Inoue Y, Nomoto A 2001 Regulation of the yeast Yap1p nuclear export signal is mediated by redox signal-induced reversible disulfide bond formation. Mol Cell Biol 21:6139–6150. doi:10.1128/mcb.21.18.6139-6150.2001. 11509657 PMC87331

[37] Delaunay A, Isnard AD, Toledano MB 2000 H_2_O_2_ sensing through oxidation of the Yap1 transcription factor. EMBO J 19:5157–5166. doi:10.1093/emboj/19.19.5157. 11013218 PMC302088

[38] Kim MS, Kim SY, Yoon JK, Lee YW, Bahn YS 2009 An efficient gene-disruption method in *Cryptococcus neoformans* by double-joint PCR with NAT-split markers. Biochem Biophys Res Commun 390:983–988. doi:10.1016/j.bbrc.2009.10.089. 19852932

[39] Kim D, Langmead B, Salzberg SL 2015 HISAT: a fast spliced aligner with low memory requirements. Nat Methods 12:357–360. doi:10.1038/nmeth.3317. 25751142 PMC4655817

[40] Pertea M, Kim D, Pertea GM, Leek JT, Salzberg SL 2016 Transcript-level expression analysis of RNA-seq experiments with HISAT, StringTie and Ballgown. Nat Protoc 11:1650–1667. doi:10.1038/nprot.2016.095. 27560171 PMC5032908

