# Supplementary figures and images for: Regulatory Mechanism of the Atypical AP-1-Like Transcription Factor Yap1 in Cryptococcus neoformans
Source: mSphere. 2019 Nov 20;4(6):e00785-19. doi: 10.1128/mSphere.00785-19 (PMC6887862; doi:10.1128/mSphere.00785-19)

**Figure S1 (So et al.)**

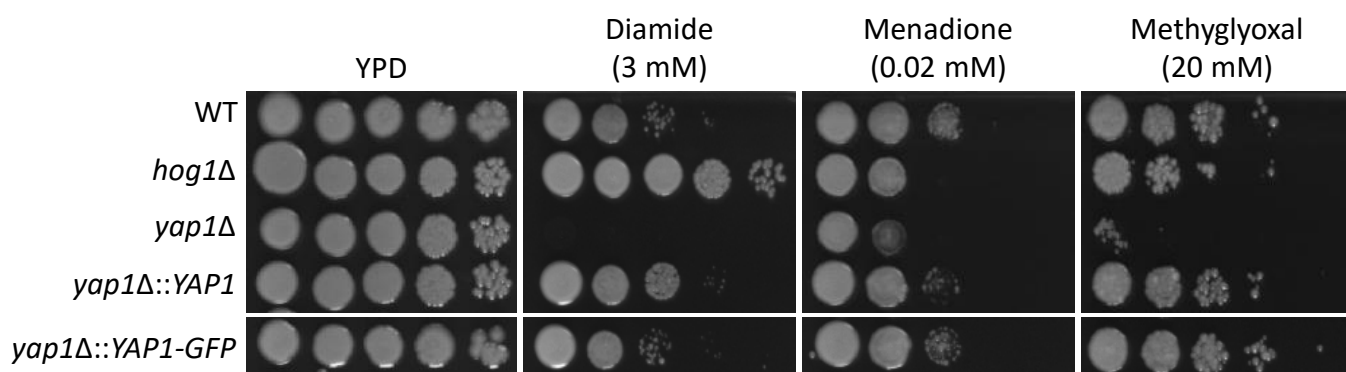

Supplement: FIG S1 [file mSphere.00785-19-sf001.pdf]

**Figure S3 (So et al.)**

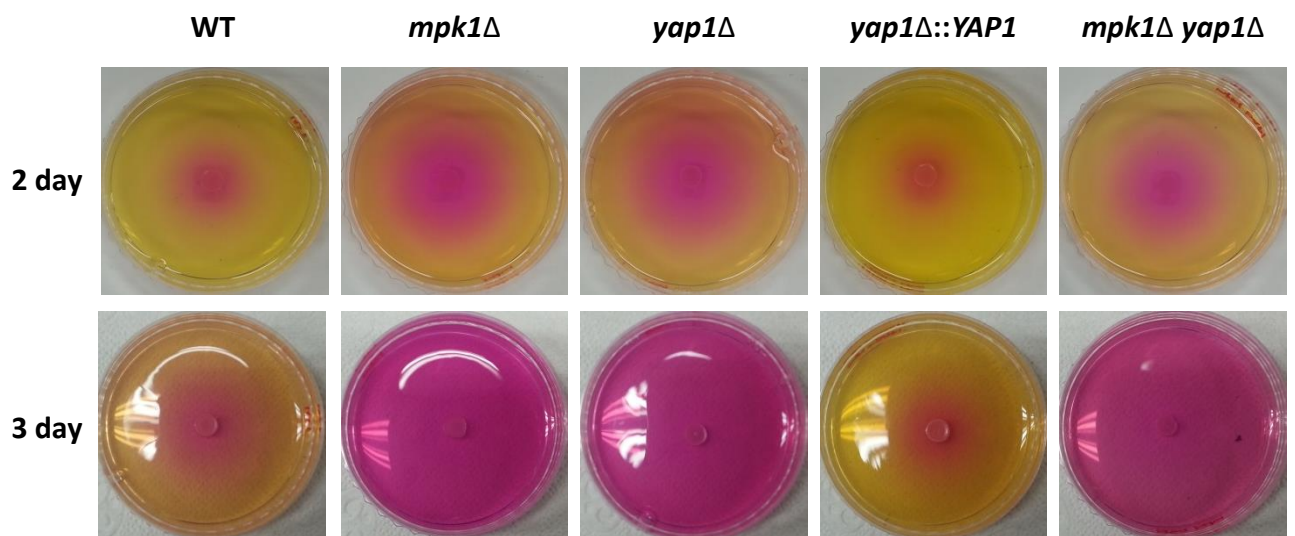

Supplement: FIG S3 [file mSphere.00785-19-sf003.pdf]
