# Supplementary material for: Regulatory Mechanism of the Atypical AP-1-Like Transcription Factor Yap1 in Cryptococcus neoformans
Source: mSphere. 2019 Nov 20;4(6):e00785-19. doi: 10.1128/mSphere.00785-19 (PMC6887862; doi:10.1128/mSphere.00785-19)
Supplement: FIG S2 [file mSphere.00785-19-sf002.pdf]

Figure S2 (So et al.)

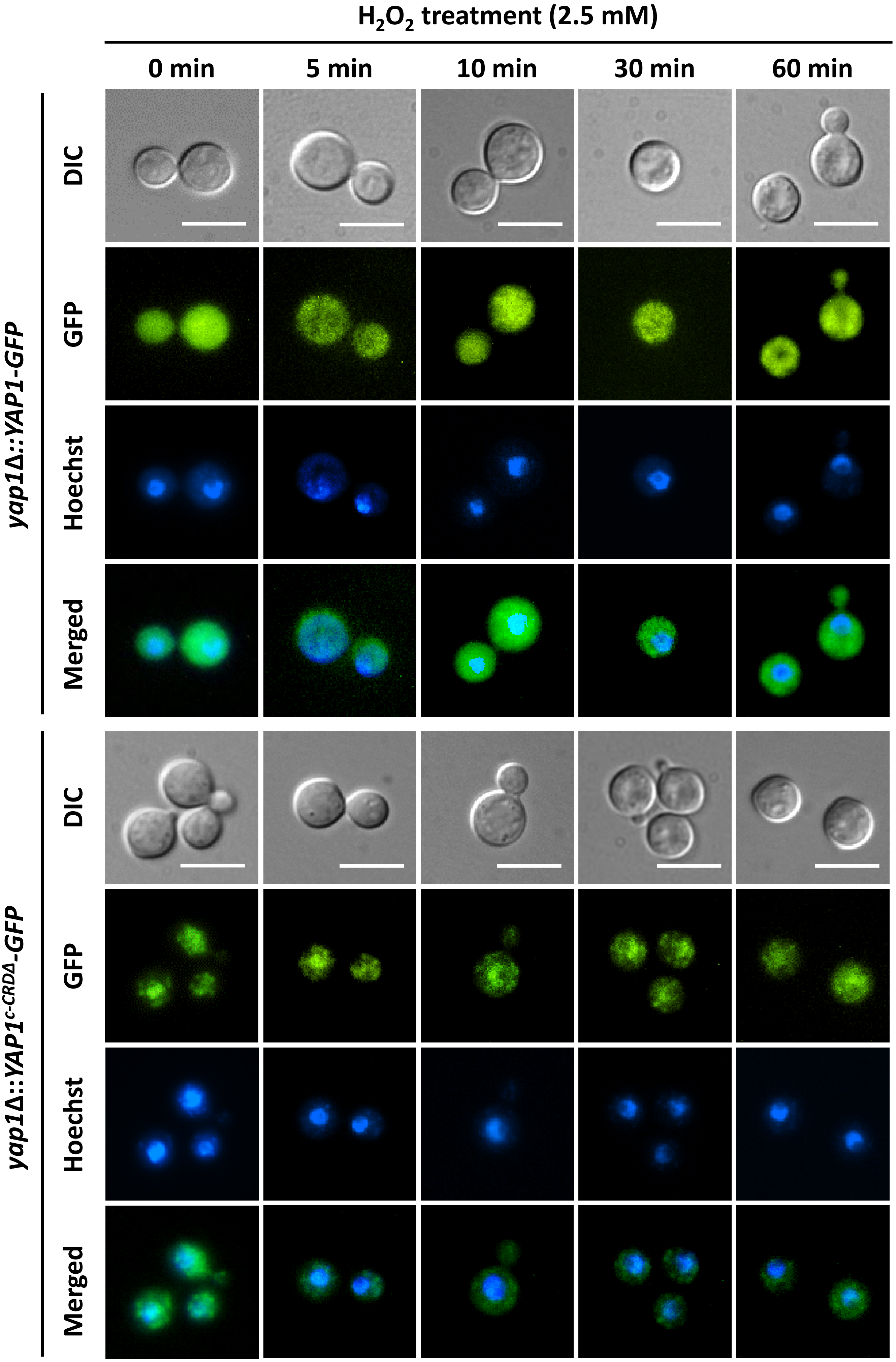

Continued

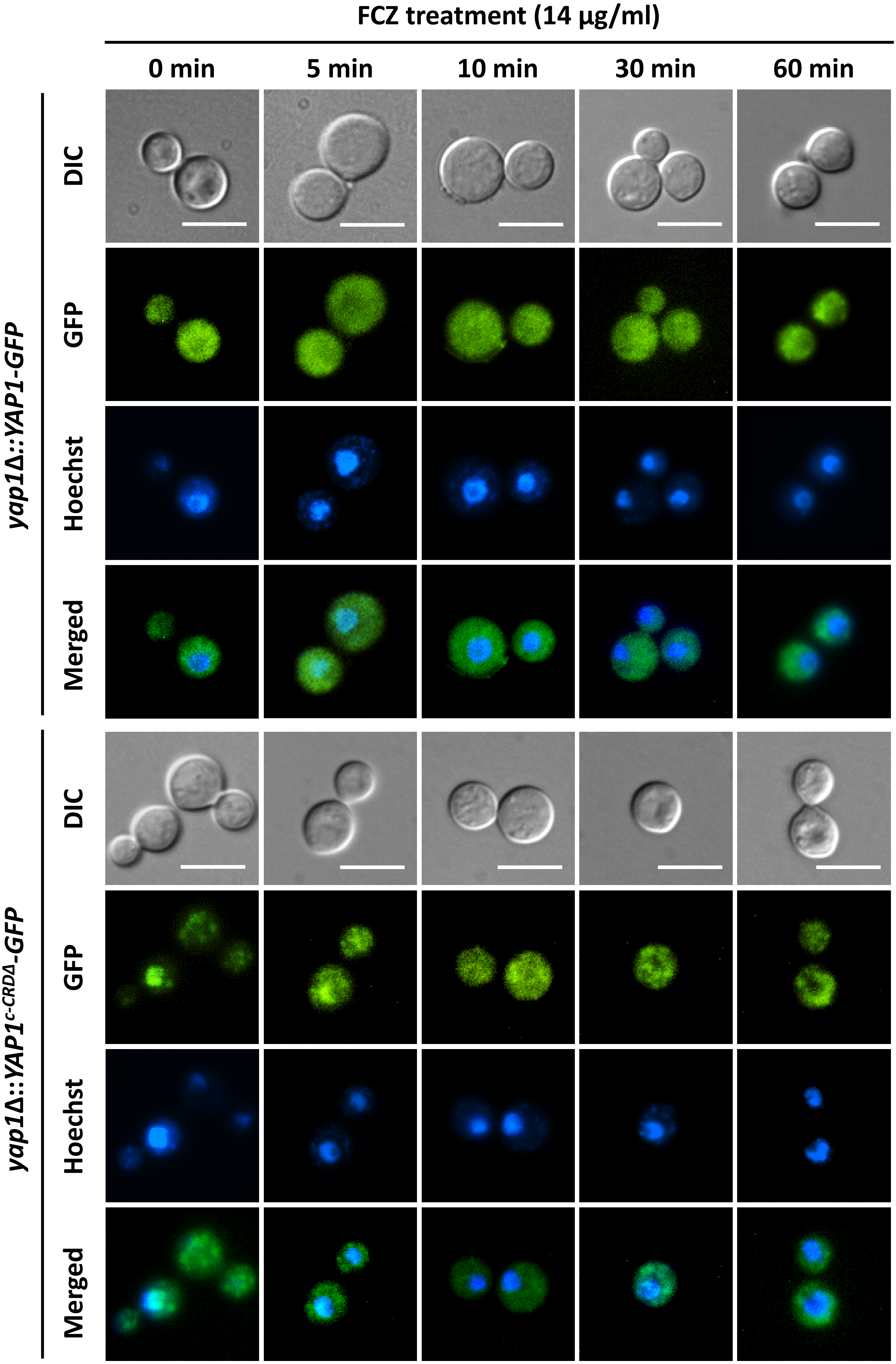

FCZ treatment (14 μg/ml)
0 min
5 min
10 min
30 min
60 min
yap1Δ::YAP1-GFP
DIC
GFP
Hoechst
Merged
yap1Δ::YAP1c-CRDΔ-GFP
DIC
GFP
Hoechst
Merged

Continued

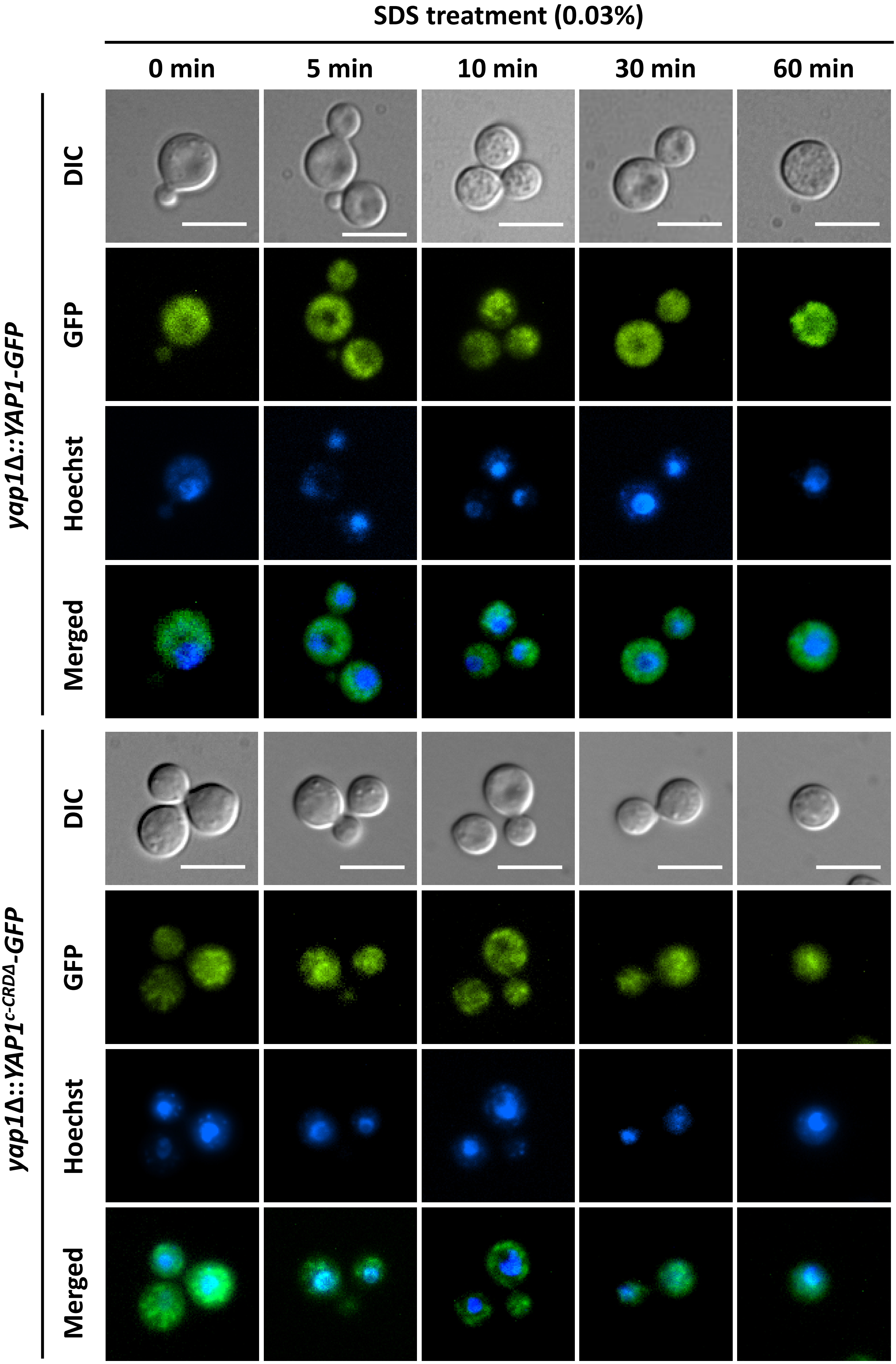

SDS treatment (0.03%)
0 min
5 min
10 min
30 min
60 min
yap1Δ::YAP1-GFP
DIC
GFP
Hoechst
Merged
yap1Δ::YAP1c-CRDΔ-GFP
DIC
GFP
Hoechst
Merged

Continued

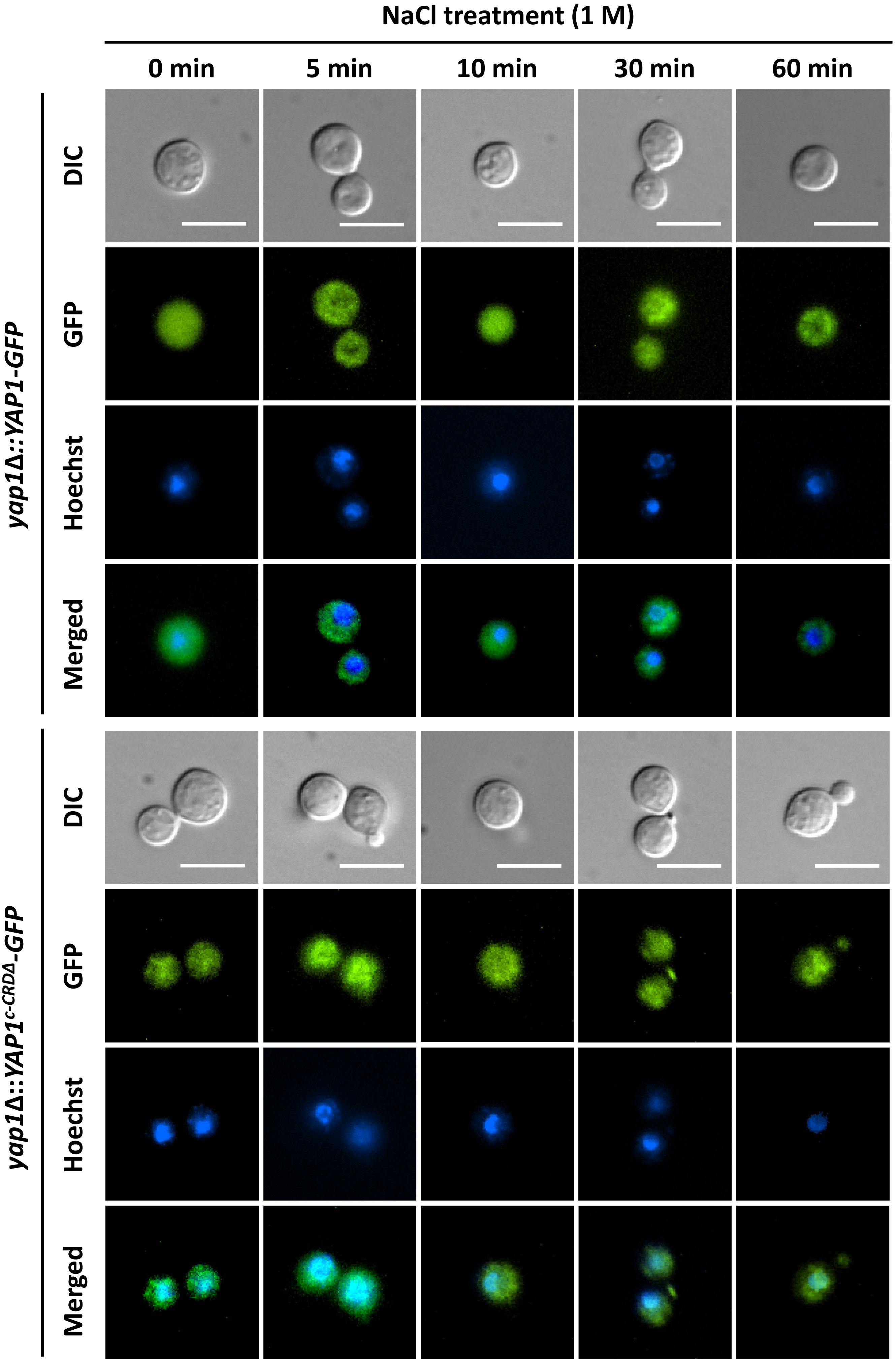

NaCl treatment (1 M)
0 min
5 min
10 min
30 min
60 min
DIC
GFP
Hoechst
Merged
yap1Δ::YAP1-GFP
yap1Δ::YAP1$^{c\text{-}CRDΔ}$-GFP

Continued

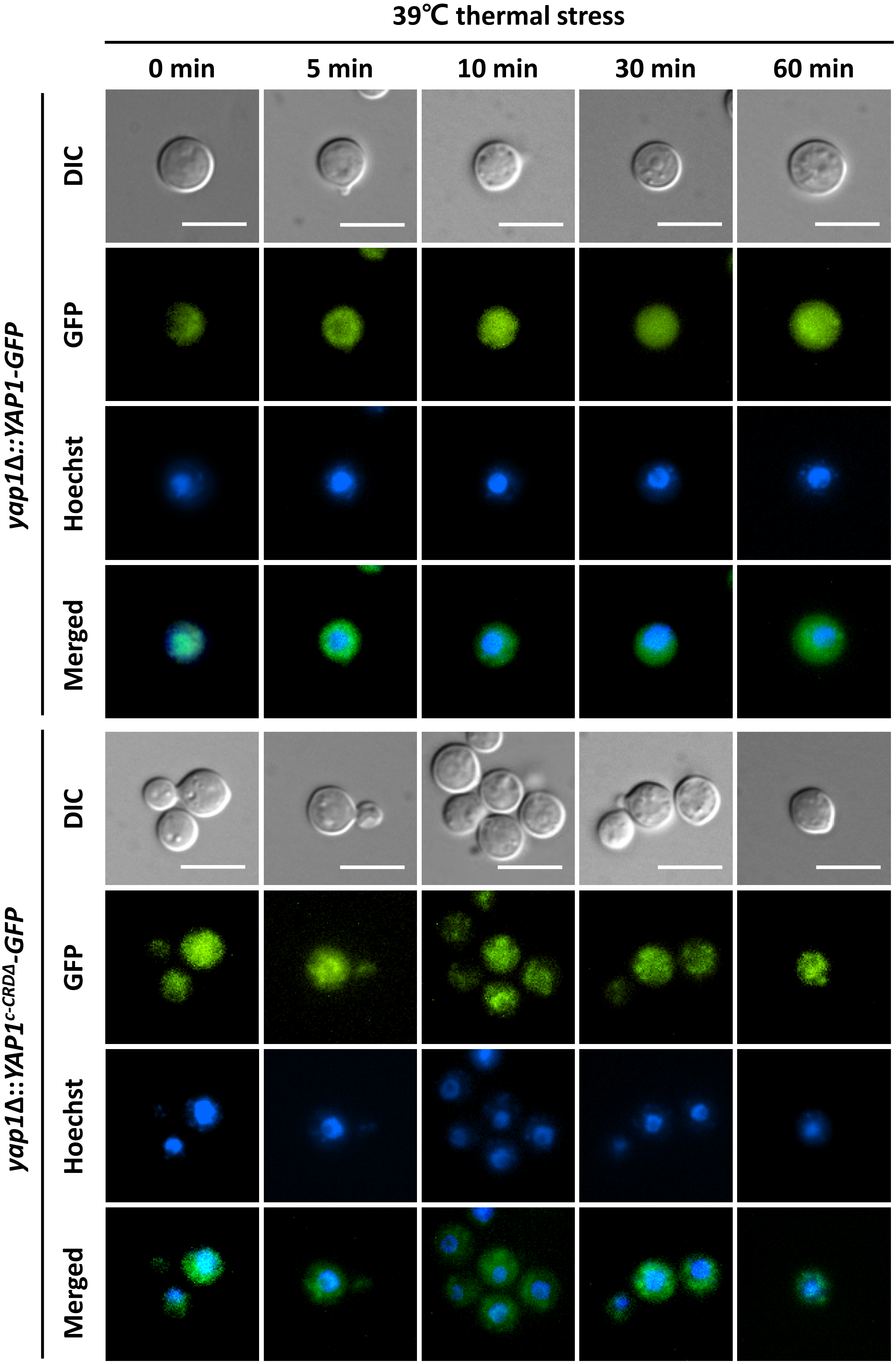
39℃ thermal stress
0 min
5 min
10 min
30 min
60 min
yap1Δ::YAP1-GFP
DIC
GFP
Hoechst
Merged
yap1Δ::YAP1c-CRDΔ-GFP
DIC
GFP
Hoechst
Merged
