# Supplementary material for: Regulatory Mechanism of the Atypical AP-1-Like Transcription Factor Yap1 in Cryptococcus neoformans
Source: mSphere. 2019 Nov 20;4(6):e00785-19. doi: 10.1128/mSphere.00785-19 (PMC6887862; doi:10.1128/mSphere.00785-19)
Supplement: TABLE S1 [file mSphere.00785-19-st001.docx]

**Table S1. *Cryptococcus* strains used in this study**

| Strain | Genotype | Parent | Reference |
| --- | --- | --- | --- |
| H99 | *MAT*α |  | ([Perfect *et al.* 1993](#_ENREF_8)) |
| KN99**a** | *MAT*a |  | ([Nielsen *et al.* 2003](#_ENREF_7)) |
| YSB64 | *MAT*α *hog1*Δ::*NAT-STM#177* | H99 | ([Bahn *et al.* 2005](#_ENREF_2)) |
| YSB815 | *MAT*α *yap1*Δ::*NAT-STM#296* | H99 | ([Jung *et al.* 2015](#_ENREF_3)) |
| YSB1290 | *MAT*α *yap1*Δ::*NAT-STM#296* | H99 | ([Jung *et al.* 2015](#_ENREF_3)) |
| YSB2122 | *MAT*α *yap1*Δ::*NAT-STM#296 YAP1*::*NEO* | YSB815 | This study |
| YSB2129 | *MAT*α *yap1*Δ::*NAT-STM#296 YAP1*::*NEO* | YSB815 | This study |
| YSB3525 | *MAT*a *yap1*Δ::*NEO* | KN99**a** | This study |
| YSB3526 | *MAT*a *yap1*Δ::*NEO* | KN99**a** | This study |
| YSB2723 | *MAT*α *yap1*Δ::*NAT-STM#296 YAP1-GFP-NEO* | YSB1290 | This study |
| YSB5796 | *MAT*α *yap1*Δ::*NAT-STM#296 YAP1^c-CRD^*^Δ^*-GFP-NEO* | YSB815 | This study |
| YSB676 | *MAT*α *atf1*Δ::*NAT-STM#220* | H99 | ([Kim *et al.* 2010](#_ENREF_5)) |
| YSB1273 | *MAT*α *tsa1*Δ::*NEO* | H99 | ([Upadhya *et al.* 2013](#_ENREF_9)) |
| YSB1274 | *MAT*α *tsa1*Δ::*NEO* | H99 | ([Upadhya *et al.* 2013](#_ENREF_9)) |
| YSB1204 | *MAT*α *tsa3*Δ::*NEO* | H99 | ([Upadhya *et al.* 2013](#_ENREF_9)) |
| YSB1205 | *MAT*α *tsa3*Δ::*NEO* | H99 | ([Upadhya *et al.* 2013](#_ENREF_9)) |
| YSB2735 | *MAT*α *tsa3*Δ::*NEO tsa1*Δ::*NAT-STM#169* | YSB1204 | ([Upadhya *et al.* 2013](#_ENREF_9)) |
| YSB2736 | *MAT*α *tsa3*Δ::*NEO tsa1*Δ::*NAT-STM#169* | YSB1204 | ([Upadhya *et al.* 2013](#_ENREF_9)) |
| YSB1667 | *MAT*α *trx1*Δ::*NAT-STM#234* | H99 | ([Upadhya *et al.* 2013](#_ENREF_9)) |
| YSB1668 | *MAT*α *trx1*Δ::*NAT-STM#234* | H99 | ([Upadhya *et al.* 2013](#_ENREF_9)) |
| YSB1791 | *MAT*α *trx2*Δ::*NEO* | H99 | ([Upadhya *et al.* 2013](#_ENREF_9)) |
| YSB1795 | *MAT*α *trx1*Δ::*NAT-STM#234 trx2*Δ::*NEO* | YSB1667 | ([Upadhya *et al.* 2013](#_ENREF_9)) |
| YSB1796 | *MAT*α *trx1*Δ::*NAT-STM#234 trx2*Δ::*NEO* | YSB1667 | ([Upadhya *et al.* 2013](#_ENREF_9)) |
| KK3 | *MAT*α *mpk1*Δ::*NAT* | H99 | ([Kojima *et al.* 2006](#_ENREF_6)) |
| YSB53 | *MAT*α *ras1*Δ::*NAT-STM#150* | H99 | ([Bahn *et al.* 2004](#_ENREF_1)) |
| YSB619 | *MAT*α *sch9*Δ::*NAT-STM#169* | H99 | ([Kim *et al.* 2009](#_ENREF_4)) |
| YSB620 | *MAT*α *sch9*Δ::*NAT-STM#169* | H99 | ([Kim *et al.* 2009](#_ENREF_4)) |
| YSB2432 | *MAT*α *yap1*Δ::*NAT-STM#296 atf1Δ*::*NEO* | YSB815 | This study |
| YSB2433 | *MAT*α *yap1*Δ::*NAT-STM#296 atf1Δ*::*NEO* | YSB815 | This study |
| YSB4949 | *MAT*α *yap1*Δ::*NAT-STM#296 atf1Δ*::*NEO* | YSB815 | This study |
| YSB4958 | *MAT*α *yap1*Δ::*NAT-STM#296 atf1Δ*::*NEO* | YSB815 | This study |
| YSB4959 | *MAT*α *yap1*Δ::*NAT-STM#296 atf1Δ*::*NEO* | YSB815 | This study |
| YSB3092 | *MAT*α *yap1*Δ::*NAT-STM#296 mpk1Δ*::*HYG* | YSB815 | This study |

Each *NAT-STM#* indicates the Nat^r^ marker with a unique signature tag

**References**

Bahn, Y. S., J. K. Hicks, S. S. Giles, G. M. Cox and J. Heitman, 2004 Adenylyl cyclase-associated protein Aca1 regulates virulence and differentiation of *Cryptococcus neoformans* via the cyclic AMP-protein kinase A cascade. Eukaryot. Cell 3**:** 1476-1491.

Bahn, Y. S., K. Kojima, G. M. Cox and J. Heitman, 2005 Specialization of the HOG pathway and its impact on differentiation and virulence of *Cryptococcus neoformans*. Mol. Biol. Cell 16**:** 2285-2300.

Jung, K. W., D. H. Yang, S. Maeng, K. T. Lee, Y. S. So *et al.*, 2015 Systematic functional profiling of transcription factor networks in *Cryptococcus neoformans.* Nat. Commun. 6**:** 6757.

Kim, M. S., S. Y. Kim, J. K. Yoon, Y. W. Lee and Y. S. Bahn, 2009 An efficient gene-disruption method in *Cryptococcus neoformans* by double-joint PCR with *NAT*-split markers. Biochem. Biophys. Res. Commun. 390**:** 983-988.

Kim, M. S., Y. J. Ko, S. Maeng, A. Floyd, J. Heitman *et al.*, 2010 Comparative transcriptome analysis of the CO2 sensing pathway via differential expression of carbonic anhydrase in *Cryptococcus neoformans.* Genetics 185**:** 1207-1219.

Kojima, K., Y. S. Bahn and J. Heitman, 2006 Calcineurin, Mpk1 and Hog1 MAPK pathways independently control fludioxonil antifungal sensitivity in *Cryptococcus neoformans*. Microbiology 152**:** 591-604.

Nielsen, K., G. M. Cox, P. Wang, D. L. Toffaletti, J. R. Perfect *et al.*, 2003 Sexual cycle of *Cryptococcus neoformans* var. grubii and virulence of congenic a and alpha isolates. Infect. Immun. 71**:** 4831-4841.

Perfect, J. R., N. Ketabchi, G. M. Cox, C. W. Ingram and C. L. Beiser, 1993 Karyotyping of *Cryptococcus neoformans* as an epidemiological tool. J. Clin. Microbiol. 31**:** 3305-3309.

Upadhya, R., H. Kim, K. W. Jung, G. Park, W. Lam *et al.*, 2013 Sulphiredoxin plays peroxiredoxin-dependent and -independent roles via the HOG signalling pathway in *Cryptococcus neoformans* and contributes to fungal virulence. Mol. Microbiol. 90**:** 630-648.
