# Supplementary material for: Regulatory Mechanism of the Atypical AP-1-Like Transcription Factor Yap1 in Cryptococcus neoformans
Source: mSphere. 2019 Nov 20;4(6):e00785-19. doi: 10.1128/mSphere.00785-19 (PMC6887862; doi:10.1128/mSphere.00785-19)
Supplement: TABLE S2 [file mSphere.00785-19-st002.docx]

Table S2. Primers used in this study

| **Primer name** | Sequence | **Comments** |
| --- | --- | --- |
| B79 | TGTGGATGCTGGCGGAGGATA | Screening primer on *ACT1* promoter |
| B1026 | GTAAAACGACGGCCAGTGAGC | M13 forward (extended) |
| B1027 | CAGGAAACAGCTATGACCATG | M13 reverse (extended) |
| B1454 | AAGGTGTTCCCCGACGACGAATCG | NSL2 |
| B1455 | AACTCCGTCGCGAGCCCCATCAAC | NSR2 |
| B1886 | TGGAAGAGATGGATGTGC | NSL-NEO |
| B1887 | ATTGTCTGTTGTGCCCAG | NSL-NEO |
| B2867 | AGGATTATCGCCTGAACC | *YAP1* L1 |
| B2868 | TCACTGGCCGTCGTTTTACATGTTGTTGAAGAGGGCG | *YAP1* L2 |
| B1589 | CATGGTCATAGCTGTTTCCTGAACGACGACCATCGCAGTAG | *YAP1* R1 |
| B2869 | CATCCCGAGTTGGAAAAC | *YAP1* R2 |
| B2870 | TGTGCCAGAAACAGTTCGC | *YAP1* SO |
| B2873 | GCTCTCTGAAAGCAAATCG | *YAP1* PO, NP1 (Southern blot, Northern blot Probe) |
| B4249 | ATGTGTGTCTCAAACCCG | *YAP1-GFP* LP |
| B4250 | CTTGGCTGCTAAAAGATTGC | *YAP1-GFP* RP |
| B3152 | \| C CAACACGGCCGCGTTCCTCG \| \| --- \| | *YAP1* SEQ1 |
| B3153 | \| T GAAGGTGATGGAAAAAGAGA \| \| --- \| | *YAP1* SEQ2 |
| B3154 | TCATCCCCTTCCATTTCC | *YAP1* NP2, SEQ3 (Northern blot Probe) |
| B3155 | ACTCTGGATGAGATTCGGG | *YAP1* SEQ4 |
| B3287 | GCCAAGGCGACGTGTAAAGAT | *YAP1* SEQ5 |
| B3288 | GCGTCTGTTATGTCCAGCTGC | *YAP1* SEQ6 |
| B8432 | gggcccATACGCCCTGTTGGTTTGC | *YAP1::YAP1^c-CRDΔ^* LP1 |
| B8433 | GAGCTCTGCCACCTTGTCCTCGAG | *YAP1::YAP1^c-CRDΔ^* RP1 |
| B8434 | GAGCTCGAAAACAAGGCATACGGC | *YAP1::YAP1^c-CRDΔ^* LP2 |
| B8435 | accggtACTATAATTCTTCGCACTG | *YAP1::YAP1^c-CRDΔ^* RP2 |
| B8436 | accggtAAAGATGGTCAGTGCTGCG | *YAP1::YAP1^c-CRDΔ^* LP3 |
| B8437 | gcggccgcCTGCGATGGTCGTCGTTCGC | *YAP1::YAP1^c-CRDΔ^* RP3 |
| B9346 | AGTCGTGGGGATGTTCTTGG | *YAP1::YAP1^c-CRDΔ^* CSO |
| B527 | AGTAGAGGAGTGGATTGGGG | *ATF1* L1 |
| B528 | CTGGCCGTCGTTTTACTGAGGCATTGAAGGAGGTGC | *ATF1* L2 |
| B7818 | CATGGTCATAGCTGTTTCCTGATGAGCGGGTAATGTCCTC | *ATF1* R1 |
| B7819 | AGAGGAACTACATACTGGGTC | *ATF1* R2 |
| B532 | AGCGGTTCGGTAGAAGACTG | *ATF1* PO |
| B531 | AAGCAAGTTGACGGCATC | *ATF1* SO |
| B5930 | TTTGCTTGCTCCTCTTCTC | *MPK1* L1 |
| B5931 | TCACTGGCCGTCGTTTTACGAGAAGTAGAGGCAGTGACG | *MPK1* L2 |
| B5932 | CATGGTCATAGCTGTTTCCTGTTGGAGAAACAGTTGGAGAG | *MPK1* R1 |
| B5933 | TTCAGCAGGTCAATCAGG | *MPK1* R2 |
| B5934 | CGACTCACGATGTAACTTCC | *MPK1* SO |
| B5935 | ACCTCAACTCTCTCAGACACC | *MPK1* PO |
| JOHE11793 | TGTGGTAGGTGCGTTATCG | *HOG1* L1 |
| JOHE11794 | CTGGCCGTCGTTTTACAGAAAGCCCATCCATCAG | *HOG1* L2 |
| JOHE11795 | GTCATAGCTGTTTCCTGTCTTGGTAAGTCTCTGTGCC | *HOG1* R1 |
| JOHE11796 | TACTCAACCCCATACTCACTCCCG | *HOG1* R2 |
| JOHE11797 | TGAAGACAAAAGGCGTGGG | *HOG1* SO1 |
| JOHE11798 | AAATCGTCCGTGAAGTCGCAGG | *HOG1* SO2 |
| JOHE11799 | TCACAGAGCGTTGATTACG | *HOG1* PO1 |
| JOHE11800 | AATCAAACACCTCGGCGGCAAC | *HOG1* P2 |

| B1697 | GGCAGGAACATCAATAATCC | *SRX1* Q-RT1 |
| --- | --- | --- |
| B6175 | GATCCTACAGGAACCCCTC | *SRX1* Q-RT2 |
| B2311 | CCATTGATACCCAAGGTCC | *SRX1* NP1 (Northern blot Probe) |
| B2312 | GGCAGGAACATCAATAATCC | *SRX1* NP2 (Northern blot Probe) |
| B1777 | AAGCACCTCCTTCAATGC | *ATF1* NP1 (Northern blot Probe) |
| B1778 | AGTAAGCCTGCCAACTTCG | *ATF1* NP1 (Northern blot Probe) |
